# Spatial immunophenotypes orchestrate prognosis in triple-negative breast cancer with Miller-Payne grade 4 following neoadjuvant chemotherapy

**DOI:** 10.1038/s41523-023-00565-8

**Published:** 2023-07-12

**Authors:** Jianli Ma, Yuwei Deng, Dawei Chen, Xiaomei Li, Zhiyong Yu, Haibo Wang, Lei Zhong, Yingjie Li, Chengqin Wang, Xiang Li, Jinming Yu, Qingyuan Zhang

**Affiliations:** 1 Department of Radiation Oncology, Shandong University Cancer Center, 440 Jiyan Road, Jinan, 250117 Shandong Province People’s Republic of China; 2 Department of Medical Oncology, Harbin Medical University Cancer Hospital, Harbin, 150081 Heilongjiang Province People’s Republic of China; 3 Department of Pathology, Harbin Medical University Cancer Hospital, Harbin, 150081 Heilongjiang Province People’s Republic of China; 4 Department of Breast Cancer Center, Shandong University Cancer Center, 440 Jiyan Road, Jinan, 250117 Shandong Province People’s Republic of China; 5 Department of Breast Disease Center, The Affiliated Hospital of Qingdao University, 16 Jiangsu Road, Qingdao, Shandong 266003 P.R. China; 6 Department of Breast Surgery, the Second Affiliated Hospital of Harbin Medical University, Harbin, 150081 Heilongjiang Province People’s Republic of China; 7 Department of Pathology, the Second Affiliated Hospital of Harbin Medical University, Harbin, 150081 Heilongjiang Province People’s Republic of China; 8 Department of Pathology, the Affiliated Hospital of Qingdao University, Qingdao, 266000 Shandong Province People’s Republic of China; 9 Department of Medical Oncology, Harbin Medical University Cancer Hospital, Heilongjiang Cancer Institute, Harbin, 150081 Heilongjiang Province People’s Republic of China

**Keywords:** Cancer microenvironment, Prognostic markers

## Abstract

Some triple-negative breast cancer (TNBC) patients evaluated as Miller-Payne 4 with ypN0 after neoadjuvant chemotherapy (NACT) who have better prognoses should avoid escalation of therapy. We aim to identify these patients by evaluating pretherapeutic spatial distributions of immunophenotypes. Our retrospective study in patients with TNBC assessed as Miller-Payne grade 4/5 with ypN0 showed that Miller-Payne 4 with ypN0 group had poorer 5-year disease-free survival (DFS, 63.8% vs. 83.0%, *p* = 0.003) and the 5-year overall survival (OS, 71.0% vs. 85.5%*, p* = 0.007) than Miller-Payne 5 with ypN0 group. High TILs were significantly associated with better DFS and OS in patients with Miller-Payne 4 and ypN0 (both *p* = 0.016). Spatially, detected by multiplexed ion beam imaging by the time of flight combined with proteomics, tumors assessed as Miller-Payne 4 and ypN0 with good prognosis exhibited an inflamed phenotype, with dominant CD8+ T cells on tumor center, few scattered CD68+ myeloid-derived cells far away from T cells, and deposit of increased activated molecules of lymphocyte. While those with poor prognoses presented excluded phenotypes, with few CD8+ T cells restricted to invasive margins and a high density of CD14^+^CD68^+^CD11c^+^ myeloid cells. A good classifier model based on 29 spatial immunophenotypes was established by the random forest algorithm (AUC = 0.975), for identifying patients with Miller-Payne 4 and ypN0 who had favorable prognoses. We also observed similar signatures in patients with Miller-Payne 5 and ypN0. Taken together, spatial immunophenotypes may assess the prognosis in TNBC patients with Miller-Payne 4 and ypN0 after NACT.

## Introduction

Triple-negative breast cancer (TNBC) lacks the estrogen receptor (ER), progesterone receptor (PR), and HER2 receptor and therefore does not respond to endocrine therapy or anti-Her2 therapy, resulting in poor prognosis^1^. Neoadjuvant chemotherapy (NACT) has been the standardized treatment for some TNBC patients, aiming at downstaging, achieving breast-conserving surgery, and monitoring treatment sensitivity for prognostic purposes^2^. Based on the Miller-Payne grading system after NACT, patients with Miller-Payne 5 and ypN0 (pathological complete remission, pCR, absence of cancer in the breast and axillary lymph nodes) and patients with Miller-Payne 4 and ypN0 (non-pCR, <10% tumor residue but absence of cancer in the axillary lymph nodes) tend to have better 5-year disease-free survival (DFS) (85% and 72%) than patients with Miller-Payne 3–1 or ypN+ (non-pCR, >10% tumor residue or residue in the axillary lymph nodes; 66%, 60%, and 55% DFS, respectively)^3^. Patients with Miller-Payne 5 and ypN0 have the highest 5-year overall survival (OS) of 95%, and patients with Miller-Payne 4 and ypN0 exhibit a favorable 5-year OS of 84.7%^4^. Notably, patients who reached pCR after NACT had a lower frequency of relapse^5^. When pCR is not reached, treatment escalation should be given to patients with the highest risk of disease progression while sparing those with good clinical outcomes^6,7^. Indeed, treatment escalation after the operation is always used in cases of residual disease as patients with Miller-Payne 4 and ypN0 in multiple clinical trials^8^, but some patients with better prognoses should avoid this escalation. The majority of studies focus on predicting the response of NACT^9^ but lack the predictors for guiding the optimal post-operation strategies to patients.

Except for the single-cell DNA- and RNA-sequencing of residual tumor cells to predict outcomes after NACT^1^, immunological parameters are potential predictors^10^. High response rates are reported to correlate with tumor-infiltrating lymphocytes (TILs) and immune-related genes in the neoadjuvant GeparSixto trial of TNBC^11^. The presence of most immunocyte types, including T cells, B cells, and myeloid-derived dendritic cells is significantly associated with a superior prognosis in TNBC. Previously, the mRNA datasets such as the MCP counter method which focuses on the absolute abundance, and the CIBERSORT method for relative quantification are the main basis for analyzing immunocyte phenotypes^12^. Currently, based on the spatial organization of tumor-infiltrating immunocytes, TNBC tumors are considered “cold,” with a low abundance of immune infiltrates, while “hot,” with a high abundance, is correlated with OS^13^. Up to now, the landscapes of spatial immunocytes in tumors with TNBC which are assessed as Miller-Payne 4 and ypN0 after NACT, and their associations with prognosis are unclear.

Here, we determine the correlation between pre-therapeutic TILs and survival of TNBC assessed as Miller-Payne 4 and ypN0 after NACT. We demonstrate that the specific spatial immunophenotypes are the potential to indicate prognosis in these patients.

## Results

### Clinicopathological characteristics have correlations with lymphocytes

TILs discovery on TNBC assessed as Miller-Payne grade 4/5 and ypN0 (*n* = 272) revealed that three categories had no difference among the NACT regimens (*p* = 0.069). Patients with Miller-Payne 5 and ypN0 tended to have higher TILs than patients with Miller-Payne 4 and ypN0 (*p* < 0.001). Patients with pretherapeutic T1 and T2 tumors accounted for 77.6% and had relatively higher TILs levels than patients with T3 tumors (*p* = 0.026). Pretherapeutic negative node status and early clinical stage (stages I and II) also tended to have more TILs infiltration (both *p* < 0.001). There was no significant association between age, menopausal status, histology or nuclear grade, and TILs (Table 1). We performed the subgroup analysis in patients with Miller-Payne 4 and ypN0 (*n* = 187) and showed that pretherapeutic node status (*p* = 0.001) and clinical stage (*p* < 0.001) were related to TILs, showing a similar trend with the whole group (Supplementary Table 1).

**Table 1:** Baseline parameters and distribution of stroma TILs in TNBC assessed as Miller-Payne 4/5 with ypN0 after NACT.

	Total		Low (0–10%)		Intermediate (11–59%)		High (≥60%)		
Variable	*n*	%	*n*	%	*n*	%	*n*	%	*P-*value
Total no.	272	100	52	19.1	131	48.2	89	32.7	–
*Age*									
≤40	107	39.3	19	17.8	48	44.9	40	37.4	0.419
>40	165	60.7	33	20.0	83	50.3	49	29.7	
*Menopausal status*									
Post	156	57.4	26	16.7	81	51.9	49	31.4	0.299
Pre/peri	116	42.6	26	22.4	50	43.1	40	34.5	
*Histology*									
Lobular and others	46	16.9	8	17.4	20	43.5	18	39.1	0.596
Ductal	226	83.1	44	19.5	111	49.1	71	31.4	
*Nuclear grade*									
1–2	141	51.8	20	14.2	71	50.4	50	35.5	0.096
3	131	48.2	32	24.4	60	45.8	39	29.8	
*Prechemotherapy tumor size*									
T1	49	18.0	9	18.4	18	36.7	22	44.9	**0.026**
T2	162	59.6	26	16.0	90	55.6	46	28.4	
T3	61	22.4	17	27.9	23	37.7	21	34.4	
*Prechemotherapy node status*									
negative	159	58.5	13	8.2	88	55.3	58	36.5	**<0.001**
positive	113	41.5	39	34.5	43	38.1	31	27.4	
*Stage (AJCC staging)*									
I	35	12.9	0	0	20	57.1	15	42.9	**<0.001**
II	150	55.1	18	12.0	76	50.7	56	37.3	
III	87	32.0	34	39.1	35	40.2	18	20.7	
*Response after NACT*									
Miller-Payne 4 and ypN0	187	68.8	46	24.6	72	38.5	69	36.9	**<0.001**
Miller-Payne 5 and ypN0	85	31.3	6	7.1	59	69.4	20	23.5	
*NACT regimens*									
Anthracycline and taxane-based	176	64.7	36	20.5	75	42.6	65	36.9	0.069
Anthracycline- based	46	16.9	5	10.9	27	58.7	14	30.4	
Other	50	18.4	11	22.0	29	58.0	10	20.0	

*χ*² test for trend. The *P*-values with statistical significance are shown in bold.

*TILs* tumor-infiltrating lymphocytes, *NACT* neoadjuvant chemotherapy.

### Prognostic values of lymphocytes in TNBC with Miller-Payne 4/5 and ypN0

**Fig. 1:**
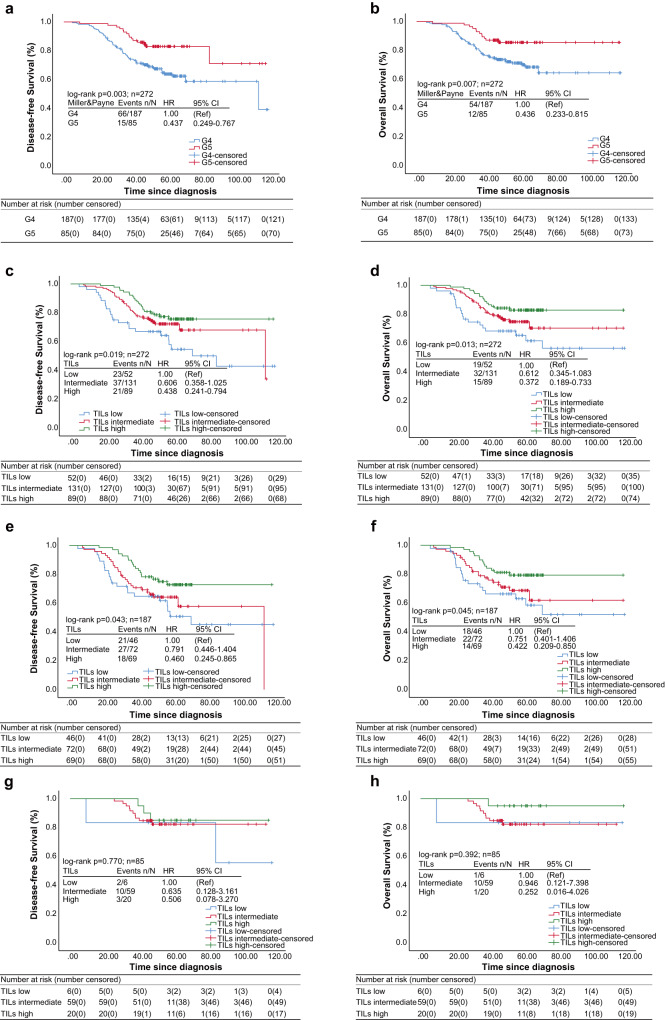
Kaplan–Meier analysis for prognosis of patients with TILs categories and Miller-Payne 4/5. **a** Disease-free survival in TNBC with Miller-Payne 4/5. **b** Overall survival in TNBC with Miller-Payne 4/5. **c** Disease-free survival in TNBC with TILs categories. **d** Overall survival in TNBC with TILs categories. Miller-Payne 4 subgroup analysis for disease-free survival (**e**) and overall survival (**f**) in TNBC patients with TILs categories. Miller-Payne 5 subgroup analysis for disease-free survival (**g**) and overall survival (**h**) in TNBC patients with TILs categories. *p-*value < 0.05 represented significance based on log-rank test. TILs tumor-infiltrating lymphocytes, TNBC triple-negative breast cancer.

During the entire group, the median follow-up was 60.3 months (range 5.73–119.83) and the 5-year OS was 74.7%. The median DFS and OS were 89.6 (95% CI 83.506–95.731) and 95.8 months (95% CI 90.410–101.214), respectively. Patients with Miller-Payne 5 and ypN0 had significantly better 5-year DFS (83.0% vs. 63.8%, *p* = 0.003) and OS (85.5% vs. 71.0%, *p* = 0.007) than patients with Miller-Payne 4 and ypN0 (Tables 2 and 3, Fig. 1a, b).

**Table 2:** Univariable and multivariable Cox regression analysis with respect to disease-free survival in all baseline parameters among the TNBC patients assessed as Miller-Payne 4/5 with ypN0 after NACT.

Patients	Number of patients *n* = 272	Number of events *n* = 81	Median DFS	95% CI	Actuarial 5-year disease-free survival (%)	Univariate analysis		Multivariate analysis	
						HR(95% CI)	*p*	HR(95%CI)	*p*
*Age, years*									
<40	107	34	89.6	(82.085–97.065)	70.7	1	0.567	–	
≥40	165	47	88.3	(78.307–98.290)	67.3	0.879 (0.565–1.367)	–		
*Histology*									
Lobular and others	46	17	83.6	(69.130–98.065)	60.1	1	0.308	–	
Ductal	226	64	91.1	(84.948–97.257)	70.6	0.758 (0.444–1.295)	–		
*Menopausal status*									
Post	156	43	89.9	(80.844–99.028)	71.9	1	0.394	–	
Pre/peri	116	38	87.9	(80.108–95.798)	64.9	1.209 (0.781–1.871)	–		
*Nuclear grade*									
1–2	141	29	99.6	(93.218–106.045)	78.1	1	**0.001**	1	**0.002**
3	131	52	80.7	(72.114–89.237)	60.0	2.132 (1.352–3.363)	2.107 (1.323–3.355)		
*Prechemotherapy node status*									
Negative	159	38	97.6	(91.270–103.868)	74.5	1	**0.010**	1	0.329
Positive	113	43	80.6	(71.167–90.072)	61.3	1.770 (1.143–2.741)	1.313 (0.760–2.268)		
*Prechemotherapy tumor size*									
T1	49	10	91.8	(84.682–98.978)	76.5	1	–	–	
T2	162	48	89.3	(81.490–97.189)	69.8	1.523 (0.770–3.014)	0.227	–	
T3	61	23	83.9	(73.227–94.563)	61.3	2.109 (1.003–4.437)	**0.049**	–	
Stage (AJCC staging)									
I	35	7	101.1	(89.148–112.989)	77.3	1	–	1	–
II	150	39	95.2	(88.381–102.101)	70.3	1.392 (0.622–3.119)	0.421	1.734 (0.744–4.040)	0.202
III	87	35	77.7	(66.955–88.562)	60.5	2.311 (1.025–5.206)	**0.043**	1.825 (0.782–4.258)	0.164
Response after NACT									
Miller-Payne 4 and ypN0	187	66	84.9	(77.806–91.980)	63.8	1	**0.003**	1	**0.005**
Miller-Payne 5 and ypN0	85	15	100.6	(91.105–110.146)	83.0	0.437 (0.249–0.767)	0.433 (0.241–0.779)		
*Stroma TILs*									
Low (0–10%)	52	23	75.1	(61.938–88.344)	54.6	1	–	1	–
Intermediate (11–59%)	131	37	89.6	(82.011–97.116)	68.9	0.606 (0.358–1.025)	0.062	0.993 (0.565–1.745)	0.980
High (≥60%)	89	21	99.5	(92.200–106.844)	75.6	0.438 (0.241–0.794)	**0.007**	0.614 (0.330–1.145)	0.125
*NACT regimens*									
Anthracycline and taxane-based	176	52	89.6	(81.914–97.381)	70.9	1	–	–	
Anthracycline- based	46	14	89.1	(77.022–101.182)	69.6	1.014 (0.562–1.830)	0.964	–	
Others	50	15	83.7	(72.929–94.534)	64.4	1.045 (0.588–1.857)	0.882	–	

The *P*-values with statistical significance are shown in bold based on the Omnibus test.

*DFS* disease-free survival, *TILs* tumor-infiltrating lymphocytes, *NACT* neoadjuvant chemotherapy, *CI* confidence interval, *HR* hazard ratio.

**Table 3:** Univariable and multivariable Cox regression analysis with respect to overall survival in all baseline parameters among the TNBC patients assessed as Miller-Payne 4/5 with ypN0 after NACT.

Patients	Number of patients *n* = 272	Number of events *n* = 66	Median OS	95% CI	Actuarial 5-year overall survival (%)	Univariate analysis		Multivariate analysis	
						HR (95% CI)	*p*	HR (95%CI)	*p*
*Age, years*									
<40	107	28	96.9	(90.660–103.250)	85.9	1	0.565	–	
≥40	165	38	92.3	(82.176–102.557)	79.4	0.866 (0.532–1.412)	–		
*Histology*									
Lobular and others	46	13	94.7	(83.094–106.392)	75.8	1	0.538	–	
Ductal	226	53	95.5	(89.463–101.641)	84.6	0.827 (0.450–1.517)	–		
*Menopausal status*									
Post	156	34	97.2	(89.824–104.507)	86.8	1	0.305	–	
Pre/peri	116	32	93.7	(86.158–101.262)	80.5	1.287 (0.794–2.085)	–		
*Nuclear grade*									
1–2	141	23	103.1	(96.972–109.221)	89.4	1	**0.001**	1	**0.003**
3	131	43	88.1	(80.143–96.093)	74.1	2.239 (1.348–3.719)	2.185 (1.307–3.654)		
*Prechemotherapy node status*									
Negative	159	32	100.6	(94.477–106.742)	88.5	1	**0.037**	1	0.656
Positive	113	34	89.5	(80.930–97.996)	78.1	1.664 (1.026–2.699)	1.133 (0.655–1.958)		
*Prechemotherapy tumor size*									
T1	49	7	85.5	(79.101–91.863)	88.2	1	–	1	–
T2	162	38	95.7	(88.612–102.863)	80.3	1.731 (0.773–3.877)	0.183	1.920 (0.833–4.427)	0.126
T3	61	21	87.6	(76.622–98.570)	75.9	2.752 (1.169–6.476)	**0.020**	2.723 (1.152–6.438)	**0.022**
*Stage (AJCC staging)*									
I	35	6	103.6	(92.182–114.997)	87.9	1	–	–	
II	150	32	98.9	(92.182–105.672)	83.1	1.277 (0.534–3.057)	0.582	–	
III	87	28	87.6	(77.858–97.313)	74.8	2.084 (0.863–5.033)	0.103	–	
*Response after NACT*									
Miller-Payne 4 and ypN0	187	54	90.7	(83.599–97.753)	71.0	1	**0.007**	1	**0.008**
Miller-Payne 5 and ypN0	85	12	106.9	(100.551–113.444)	85.5	0.436 (0.233–0.815)	0.411 (0.213–0.790)		
*Stroma TILs*									
Low (0–10%)	52	19	82.6	(69.489–95.829)	66.5	1	***–***	1	**–**
Intermediate (11–59%)	131	32	94.8	(86.917–102.810)	78.8	0.612 (0.345–1.083)	0.092	0.867 (0.478–1.575)	0.640
High (≥60%)	89	15	104.9	(98.287–111.542)	87.3	0.372 (0.189–0.733)	**0.004**	0.468 (0.233–0.941)	**0.033**
*Neoadjuvant therapy*									
Anthracycline and taxane-based	176	40	97.3	(90.663–104.006)	84.7	1	**–**	–	
Anthracycline- based	46	12	94.9	(82.846–106.974)	80.8	1.117 (0.586–2.129)	0.737	–	
Others	50	14	84.585	(73.451–95.719)	75.4	1.260 (0.685–2.317)	0.457	–	

The *P*-values with statistical significance are shown in bold based on the Omnibus test.

*OS* overall survival, *TILs* tumor-infiltrating lymphocytes, *NACT* neoadjuvant chemotherapy, *CI* confidence interval, *HR* hazard ratio.

The univariable analysis showed TILs category and Miller-Payne 4/5 and ypN0 were associated with DFS and OS. For multivariate analysis, TILs had no significant independent associations for DFS (intermediate, HR = 0.993, 95% CI 0.565–1.745, *p* = 0.980; high, HR = 0.614, 95% CI 0.330–1.145, *p* = 0.125) and OS (intermediate, HR = 0.867, 95 % CI 0.478–1.575, *p* = 0.640; high, HR = 0.468, 95% CI 0.233–0.941, *p* = 0.033). Indeed, Miller-Payne 4 and ypN0 was a negative predictor for DFS (HR = 0.433, 95 % CI 0.241–0.779, *p* = 0.005) and OS (HR = 0.411, 95 % CI 0.213–0.790, *p* = 0.008) compared with Miller-Payne 5 and ypN0 (Tables 2 and 3). Analysis in the subgroup of Miller-Payne 4 and ypN0 indicated that high TILs yielded better DFS (HR = 0.518, 95% CI 0.274–0.979; *p* = 0.043) and OS (HR = 0.475, 95% CI 0.235–0.963; *p* = 0.039) than low TILs (Supplementary Tables 2 and 3).

### Effect of lymphocytes on survival in TNBC patients with Miller-Payne 4/5 and ypN0

Among the whole group, the 5-year DFS in patients with high TILs was 75.6% and 5-year OS was 87.3%, which were significantly better than that in patients with low TILs (Tables 2 and 3). The high TILs levels were associated with significantly better DFS (*p* = 0.019) and OS (*p* = 0.013) than low TILs levels (Fig. 1c, d).

Subgroup analyze in patients with Miller-Payne 4 and ypN0 showed that the high TILs group had significantly better survival than the low TILs group (*p* = 0.043 for DFS and *p* = 0.045 for OS, Fig. 1e, f). However, subgroup analyze in patients with Miller-Payne 5 and ypN0 showed that there was no significant survival difference among TILs categories (*p* = 0.770 for DFS, *p* = 0.392 for OS, Fig. 1g, h), consistent with previous study^12^.

### Distributions of pretherapeutic spatial immunophenotypes indicate prognosis

**Fig. 2:**
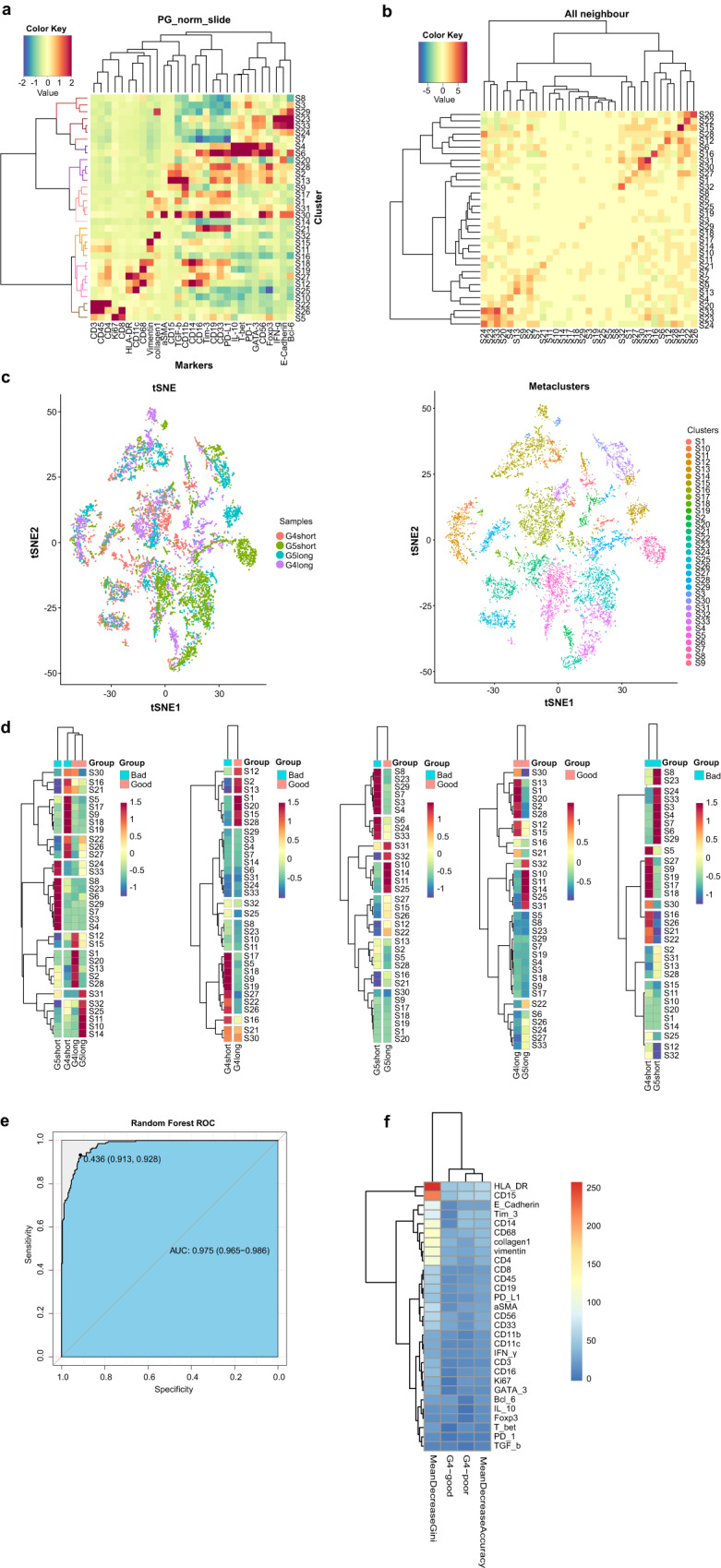
Different spatial distributions of various immunophenotypes were associated with diverse prognoses in Miller-Payne 4/5 patients. **a** Immunophenotypes from all regions were standardly clustered by protein expression markers (total 33). Scaled from −2 to 2. **b** Heatmaps denote spatial proximity *z* scores between pairs of clusters (*y*-axis) in regions of all neighbors, scaled from −5 to 5. **c** *t-SNE* plots showed the similarity of immunophenotypes between compared groups (left), and divided immunophenotypes population according to clusters (right). **d** The different expression values of 33 resulting clusters (*y*-axis) between compared groups (*x*-axis) are shown, scaled from −1 to 1. **e** The random forest classifier assessed by receiver operating characteristic (ROC) curve with the area under the curve (AUC). **f** Heatmaps character the spatial distinction of immunophenotypes between Miller-Payne 4 groups with different prognoses. t-SNE t-distributed stochastic neighbor embedding. G4 Miller-Payne 4, G5 Miller-Payne 5.

**Fig. 3:**
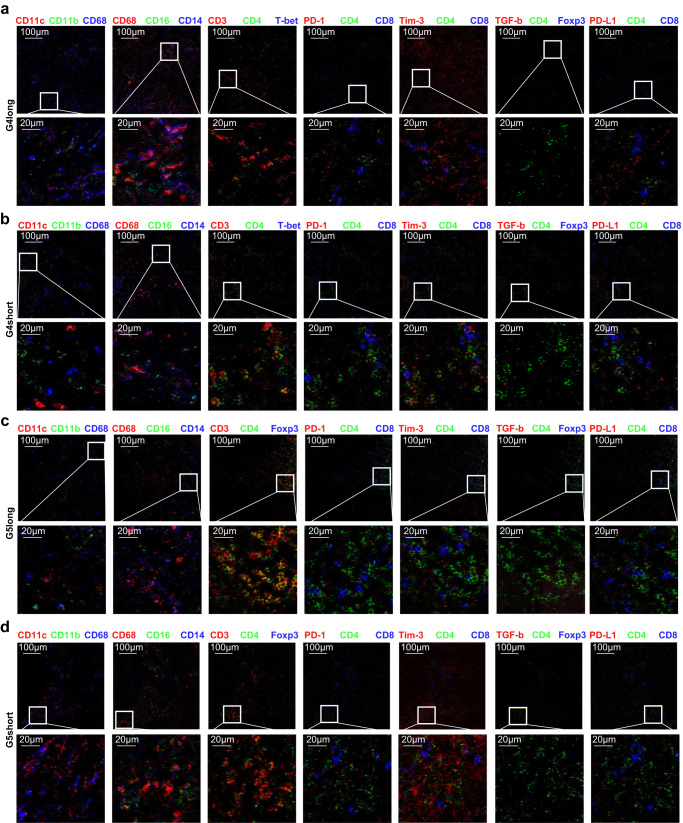
The different spatial distributions of immunophenotypes in Miller-Payne 4/5 groups associated with good and poor prognosis. **a** for Miller-Payne 4 with good prognosis. **b** for Miller-Payne 4 with poor prongosis. **c** for Miller-Payne 5 with good prognosis. **d** for Miller-Payne 5 with poor prongosis. Upward side, 500 μm × 500 μm region, scale bar: 100 μm; Downward side, 100 μm × 100 μm, scale bar: 20 μm. G4 Miller-Payne 4, G5 Miller-Payne 5.

In the pretherapeutic tumor stroma, the spatial distance between immunophenotypes were evaluated in identified 33 clusters (Fig. 2a, b). In groups with good survival, Miller-Payne 4 and ypN0 group had a majority of similar immunophenotypes clusters with Miller-Payne 5 and ypN0 group. While in groups with poor survival, Miller-Payne 4 and ypN0 group had more separated clusters with Miller-Payne 5 and ypN0 (Fig. 2c, d). The Miller-Payne 4 and ypN0 group with good prognosis exhibited an inflamed phenotype. There was abundant focal infiltration of T-cells adjacent to the tumor cells with high Ki67 (S7, S8, and S9), which co-localized with tumor PD-L1/PD-L2. These were mainly activated CD8^+^ T cells (mostly PD-1^−^IFN-γ^+^HLA-DR^+^) (S2 and S8). Scattered suppressive Tim-3^+^ CD8^+^ T-cells (S7) were found at the tumor border. The CD4^+^ cohorts, including effective Th1 (CD3^+^CD4^+^T-bet^+^) and Th2 (CD3^+^CD4^+^GATA3^+^) cells (S6 and S8), and a few CD4^+^ Foxp3^+^ Tregs (S7 and S8), were also found sporadically and adjacent to CD8^+^ T cells. Few NK cells and sporadic CD19^+^ B cells were at the tumor border. Among the small number of myeloid-derived cells (S2 and S15), stromal rather than intratumoral CD14^+^CD68^+^CD11c^+^monocytes/macrophages (M1-like) expressed lower TGF-β (S6). Few immunosuppressive CD15^+^ granulocytes (S6) were found around collagen agglomerations in the stromal region. There were few vascular endothelial cells and fibroblasts in the border regions, indicating the incapacity for invasion. In striking contrast, Miller-Payne 4 and ypN0 group with poor prognosis presented an immune-excluded phenotype. The distance between rare infiltrating lymphocytes (mainly CD8^+^ T cells) and tumor cells was significantly larger, where lymphocytes were restricted to the tumor margin. The dominant myeloid cells were scattered, separated, and isolated from tumor cells. Sporadic Tregs and PD-1^+^Tim-3^+^IFN-γ^+^HLA-DR^+^ T cells were in contact with tumor PD-L1/PD-L2 to promote immune escape. Massive Ki67 and α-SMA staining in the border regions indicated highly invasive tumors (Fig. 3a, b; Supplementary Figs. 1a, b and 2a–f). Besides, these spatial immunophenotypes could distinguish Miller-Payne 4 and ypN0 subgroups with different prognoses (Random forest algorithm, AUC = 0.975, Fig. 2e). The values of mean Decrease Gini and mean Decrease Accuracy indicated the importance (weight) in making the prediction^14^. Totally 29 immunophenotypes with their distribution features were identified and formed a good classifier panel (Fig. 2f).

The Miller-Payne 5 and ypN0 group with favorable outcomes (low Ki67; S1) also had an inflamed phenotype. A high number of tertiary lymphoid structures (TLS) were aggregated at the tumor border. The myeloid cells in the stroma region (S15 and S9) were CD68^+^ with low TGF-β and IL-10 levels (S1). Around the tumor center, the dense CD8^+^T cells, including a low number of the PD-1^+^Tim-3^+^IFN-γ^+^HLA-DR^+^ subtype, were adjacent to CD4^+^ T-cells of active Th1 and Th2 subtypes (S14), the rare Tregs and Bcl-6^+^ follicular T helper cells (Tfh). The isolated CD11b^+^CD15^+^ granulocytes (S11) presented scattered distribution. The Miller-Payne 5 and ypN0 group with inferior survival showed a relatively inflamed phenotype with focal infiltration of lymphocytes. There was a certain abundance of TLS at the tumor border. There were more CD8^+^ than CD4^+^ T cells, and both were predominantly PD-1^−^Tim-3^+^IFN-γ^−^HLA^−^DR^−^, distant from the PD-L1/PDL2 locus. Sporadic Tregs and aggregated CD68^+^CD11c^+^ macrophages which were separated from T cells were at the border regions (Fig. 3c, d; Supplementary Fig. 1c, d and Supplementary Fig. 2g–l).

Taken together, the pretherapeutic spatial immunophenotypes had prognostic associations in TNBC assessed as Miller-Payne 4 and ypN0 after NACT.

### Patterns of immunophenotyping molecules indicate the activated status of spatial immunocytes

**Fig. 4:**
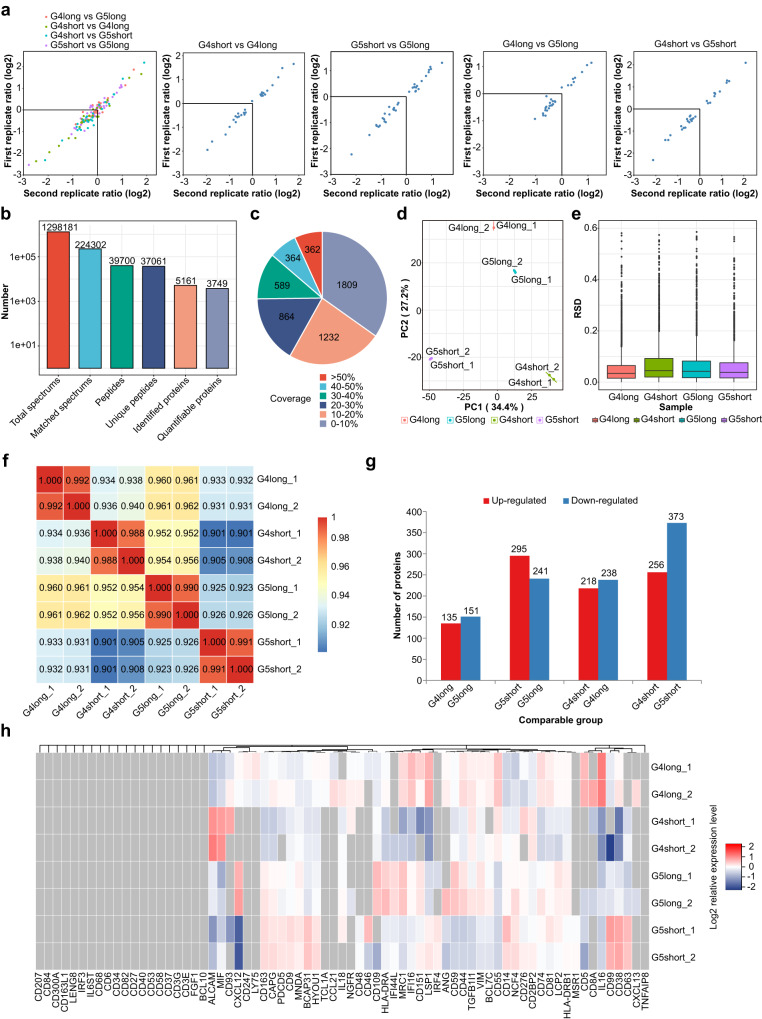
Different expression of immunophenotype proteins between Miller-Payne 4 and 5 groups with distinct prognoses. **a** Similar dispersion for biological replications. **b** Identification of 3749 different quantifiable proteins. **c** Distribution of proportions associated with differentially expressed proteins. **d** Principal component analysis (PCA) showed a high difference between compared groups and a low variation degree between the samples. **e** Box plots showed relative standard deviation (RSD) where low RSD means good quantitative repeatability. **f** Pearson’s correlation coefficient suggested the degree of correlation. Range from −1 to 1. **g** Total number of differentially expressed proteins. **h** Hierarchical clustering of the typically different immunophenotypes. G4 Miller-Payne 4, G5 Miller-Payne 5.

**Fig. 5:**
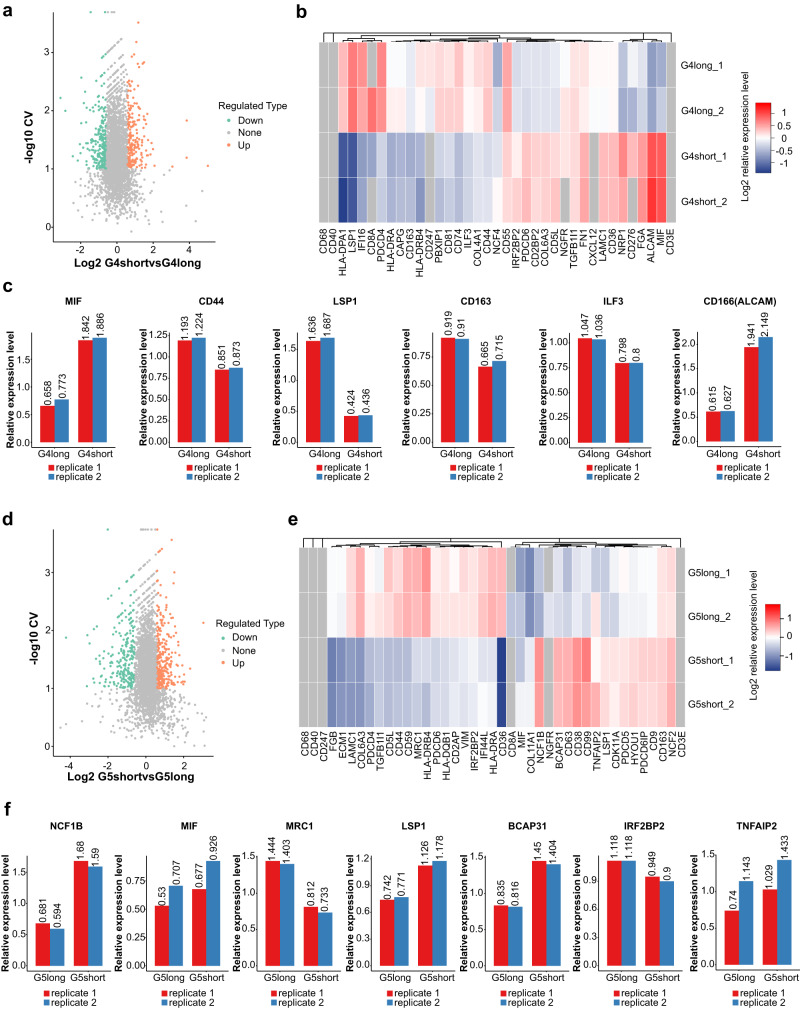

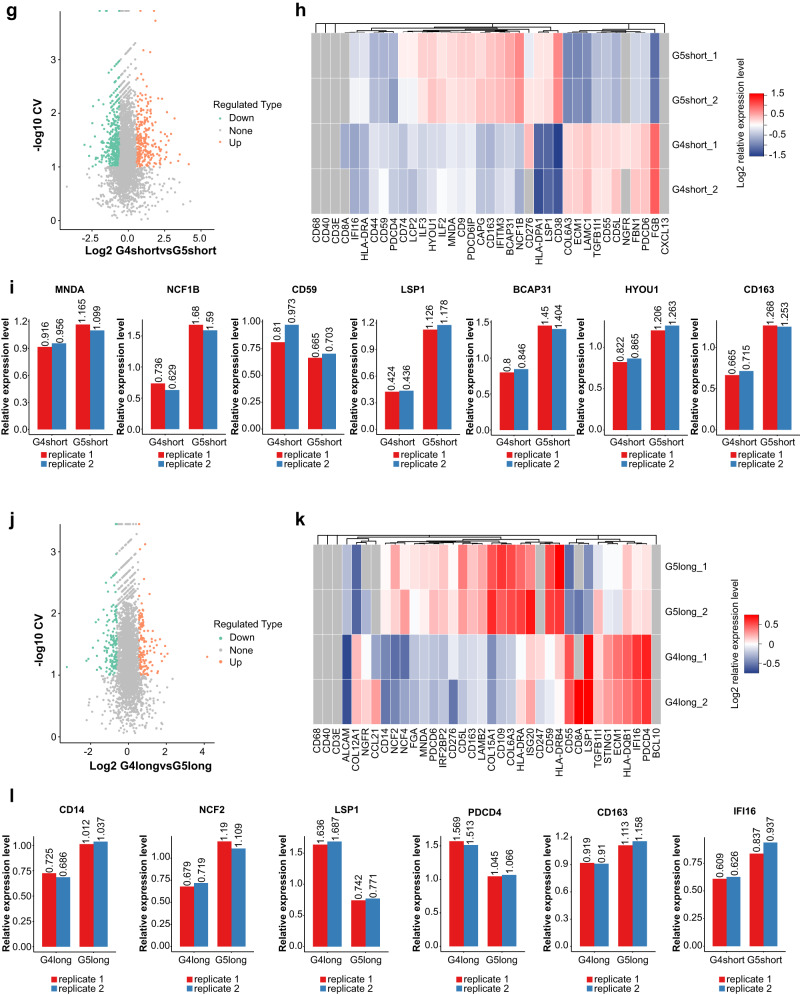
Identification of different immunophenotype proteins associated with distinct prognosis in Miller-Payne 4/5 cohorts. **a**, **d**, **g**, **j** Volcano plot shows significantly altered proteins with a fold change >1.2. Samples are compared by coefficient of variation (CV), and values <0.1 were considered significant. **b**, **e**, **h**, **k** Heat map shows levels of differentially and similarly immune-related protein. **c**, **f**, **i**, **l** Expression level of representative protein with a significant difference, quantified using Log2 ratio. G4 Miller-Payne 4, G5 Miller-Payne 5.

Totally 3749 quantified proteins were identified (Fig. 4a–c and Supplementary Fig. 3) with statistical consistency (Fig. 4d–f). Representative differentially abundant immunophenotypes such as CD8, CD14, and CD163, were similar between Miller-Payne 4 with ypN0 and Miller-Payne 5 with ypN0 group. Notably, in the subgroup of Miller-Payne 4 and ypN0, 109 significantly upregulated immune-activated components and 76 significantly downregulated immunosuppressive proteins were identified in those with good prognosis than those with poor survival (partial show, Fig. 4g, h). The marker proteins related to lymphocytes activation (e.g. LSP1, IFI16) dominated in those with favorable prognosis, while myeloid immune molecules (e.g. TGFB1I1, CD163, CAPG) increased in those with poor survival. The subgroup of Miller-Payne 5 and ypN0 also exhibited a similar pattern to that of Miller-Payne 4 and ypN0. Moreover, tumors with Miller-Payne 5 and ypN0 always exhibited a higher proportion of both lymphoid (LSP1, TNFAIP2, BCAP31) and myeloid (MIF, MCR1, TGFB1I1) immunophenotypes proteins than tumors with Miller-Payne 4 and ypN0 (Fig. 5).

We enriched five profiles stratified by prognosis in Miller-Payne 4/5 with ypN0 (Supplementary Figs. 4 and 5). These profiles were associated with different frequencies of major activated T cells (mainly CD8^+^ T cell markers), M1- and M2 macrophages, as well as collagens. Specifically, patterns 1–3 associated with immune biological processes, cellular components, and molecular functions might imply more active immune cell processes, including T and B cells regulation, chemotaxis, and IFN-γ response, in the cohort with good prognosis compared to the cohort with poor prognosis, but they relatively decreased in Miller-Payne 4 with ypN0 compared to Miller-Payne 5 with ypN0. After testing the three patterns in groups with similar survival, the immune activities (e.g. T and B cell-mediated immunity, interleukin regulation) tended to decrease in Miller-Payne 4 with ypN0 compared to Miller-Payne 5 with ypN0 (Supplementary Figs. 6 and 9a). Pattern 4 consisted of functional pathways complementing the various interactions between proteins related to T cell activation, phagocytosis, and TGF-β signaling (Supplementary Figs. 7 and 9b). Pattern 5 enhanced the crosstalk with the inflamed phenotypes acting as effectors of antigen transporter, scavenger receptor, and HLA-II recognition (Supplementary Figs. 8 and 9c). Miller-Payne 4 and ypN0 with good prognosis had more inflammation-related sponsors such as T and B cell regulation, cytokine response and cell surface signaling than those with poor survival (fold change cutoff of 1.5) (Supplementary Fig. 10). It stood to reason that Miller-Payne 5 with ypN0 also had a similar trend and exhibited enhanced TNF-signaling, IFN, antigen processing and presentation, T cell co-activation or co-inhibition and phagocytosis compared to Miller-Payne 4 with ypN0.

## Discussion

Here we report that specific spatial immunophenotypes are able to act as predictors to identify some patients assessed as Miller-Payne 4 and ypN0 who have a good prognosis for avoiding intensive therapy^15^, based on the correlation between spatial immunophenotypes and the prognosis and response to anti-PD-1 therapy^16^. In clinical pathology, we focus on stromal TILs counts as prognostic factors^17^. We find that tumors assessed as Miller-Payne 4/5 with ypN0 after NACT have a consistent positive association with increased TILs as previously^18,19^. We observe that increased TILs are linked to longer DFS and OS than that to low TILs, which is also investigated by multiple clinical trials^20,21^, with only a few exceptions^22^. TILs are associated with survival in patients with Miller-Payne 4 and ypN0 (non-PCR), but once pCR (Miller-Payne 5 and ypN0) is reached, TILs are no longer linked to DFS and OS, which is ascribed to the significant association of TILs with incremental pCR^12^. Factors that affect infiltration of TILs^23^ like nuclear grade, also have prognostic significance. Meaningfully, we find that TILs are not an independent prognostic factor for the whole group, but have prognostic significance in Miller-Payne 4 with ypN0 subgroup^24^. This provides the necessity to extend spatial phenotype-specific classifications mode among patients with Miller-Payne 4 and ypN0.

Multiple studies use single-cell genomic analysis^25^ to examine the relative abundance of immunocytes^26^, while ignoring their spatial distributions which could indicate the complicated interactions among the immune responses and tumor heterogeneity^13^. Other study defines an immune-desert phenotype with a deficiency of active immunocytes, immune-excluded tumors containing immune barriers, and inflamed tumors with effective antitumor immune cells^27^. Based on gene profiling, four distinct TME signatures are identified as “Immune desert” 8 (ID), “Margin restricted (MR)”, “Fully inflamed” (FI) and “Stroma restricted” (SR)^28^. Referring to these pioneers, we novelly perform MIBI-TOF^16^ to classify spatial immunophenotypes in situ. Notably, the inflamed phenotype in the subgroup of Miller-Payne 4/5 with ypN0 who have good prognosis is characterized by CD4^+^ and CD8^+^ T cells^29^ that are preferentially adjacent to tumor cells. The exclusion of B-cells and macrophages at the tumor border while the recruitment of intratumoral CD8^+^ T cells suggests enhanced anti-tumor immunity. Another study postulates that the inflamed phenotype could reactivate IFNγ and TNFα while omitting TGFβ and IL-10^30^. Specifically, the high frequencies of activated T-cells in the tumor center and recruitment of minor monocytes located at the border are part of a positive-feedback loop for the initial immune response^31^ in Miller-Payne 4 and ypN0 with good prognosis. Interestingly, a spatial neighbor relationship between T cells and myeloid cells in Miller-Payne 4 with ypN0 demonstrates that proximity to tumor cells is a prerequisite for the effective antitumor activity of lymphocytes, which is related to prognosis. We establish a good classifier model by integrating significant spatial immunophenotypes, hoping to identify Miller-Payne 4 and ypN0 patients with different prognoses.

Besides, the inflamed phenotype has a high expression of patterns associated with T-cell activation and interleukin-mediated signaling, including significant Th1 and Th2 differentiation and TNF signaling, which are interrelated and most likely represent upstream regulators that contribute to T-cell infiltrations. Miller-Payne 4/5 with ypN0 patients with better survival exhibits strong correlations with T cell receptor signaling pathways and the abundance of CD8^+^ T cells. We also observe an inverse correlation between TGFβ signaling activity and the presentation of T-cell infiltration in Miller-Payne 4 with ypN0, which exhibits an immune-excluded phenotype. It proves that the T lymphocytes in tumors assessed as Miller-Payne 4 with ypN0 are subjected to relatively immune suppression or not competent. The inflamed phenotype has the highest expression of proteins associated with IFN, interleukin, and chemo-attractants. The excluded phenotypes as observed in Miller-Payne 4 and ypN0 with poor prognosis have a high expression of collagen, which implies a physical barrier against T-cell infiltration^32^. In addition, the tumor progression and infiltrations of lymphocytes are inter-related^33^. For example, Miller-Payne 4 with ypN0 exhibiting better clinical outcomes has increased antigen presentation and interferon signaling^34^, associated with downregulated PI3K-AKT signaling.

At present, the clinicopathological assessment of prognosis through TILs counts before NACT is largely limited in accuracy. Although a single-cell sequencing methodology can profile the various immunocytes, it is expensive and difficult to popularize and lacks information on spatial distributions of immune cells^16^. It is a prerequisite for effective anti-tumor immunity that tumor cells are adjacent to activated T cells and far away from myeloid cells. We expect to match these images with corresponding clinicopathological sections and reproduce these spatial phenotype-specific features of tumor-infiltrating immunocytes by immunohistochemical examination of successive pathological sections. Spatial immunophenotype features contribute to identifying patients assessed as Miller-Payne 4 with ypN0 who have a better prognosis and should avoid escalation of therapy. This prognostic evaluation mode needs to be formulated in detail and verified in prospective studies as well as long-term follow-up.

## Methods

### Patients

Totally 272 patients were retrospectively reviewed and confirmed the diagnosis by experienced pathologists between January 2010 and January 2020 from four clinical centers including the Shandong Cancer Hospital affiliated to Shandong University, the Harbin Medical University Cancer Hospital, the Secondary Affiliated Hospital of Harbin Medical University, and the Affiliated Hospital of Qingdao University. The study was performed in line with the Declaration of Helsinki and ethics approval was received from the institutional review board of these clinical centers (KY2020-11). All participants provided written informed consent. The prechemotherapy node-negative status was first dependent on clinical physical examination and ultrasound. For suspicious lesions/samples, hollow needle puncture or fine needle aspiration were conducted for pathological confirmation. Lacking more than 90% of tumor cells was defined as Miller-Payne 4 and no malignant cells identifiable in sections was Miller-Payne 5^35^. The clinicopathological features are shown in Tables 1 and S2.

### Pathologic assessment

Stromal TILs were evaluated on H&E sections from core biopsies obtained before NACT. TILs were assessed according to the criteria from the International Immuno-Oncology Biomarker Working Group. The infiltrations of TILs were classified into three categories as follows: low TILs (0–10%), intermediate TILs (11–59%), or high TILs (60–100%), as previous study^12^.

### Multiplexed ion beam imaging by the time of flight (MIBI-TOF)

Slides were stained with the primary antibodies (Supplementary Table 4) with metal-conjugation by the application of the Maxpar labeling kit (Fluidigm) using concentrations based on BioTek (Berten Instruments) at 0.5 g/L. Sections were assessed by pathologists to determine the tumor boundary based on H&E staining. After de-waxing, samples should receive antigen retrieval (R&D systems) and blocking. The antibody cocktail was added to the samples overnight at 4 °C. The samples were then stained using Intercalator-Ir (Fluidigm, 201192A) for detecting DNA. Based on H&E staining, scanning was conducted on appropriate 500 × 500 µm sites through MIBI-TOF Imaging System (Fluidigm) at 200 Hz with interspersed acquisition of isotope polymers (Fluidigm) for monitoring the stability. MCDViewer, CellProfiler, and HistoCAT were applied to process image captures. The data were divided into single cells by CellProfiler (v3.1.8.) according to the Fluidigm DNA markers and cell membranes (e.g., CD3, CD4, or CD8). The single-cell markers were quantified by histoCat v1.75. The standardized data were processed through Harmony in t-distributed stochastic neighbor embedding (*t*-SNE) and PhenoGraph analysis. PhenoGraph (v.2.0) was applied for the aggregation of subgroups based on their markers. The neighbor interactions of cell types with enrichment or depletion were detected through the CellProfiler Measure Object Neighbors module and neighborhood (https://github.com/BodenmillerGroup/neighbouRhood), combined with permutation-test-based analysis of the spatial distribution. Four pixels (4 µm) were used to define the boundary between neighboring cells. Significant differences were identified as *p*-value < 0.01.

### 4D label-free proteomics

The tryptic peptides were loaded onto a homemade reversed-phase analytical column (25 cm length, 75 μm i.d.) through a nanoElute UHPLC system (Bruker Daltonics). The separated peptide peaks were injected into a capillary plasma source, which was connected to a timsTOF Pro (Bruker Daltonics) mass spectrometry instrument operated in parallel accumulation serial fragmentation (PASEF) mode. The MaxQuant search engine (v.1.6.6.0) was used for processing the resulting MS/MS data. Tandem mass spectra were searched based on the Homo_sapiens_9606_SP_20191115 database (20,380 sequences) concatenated with a reverse decoy database. Trypsin/P was indicated as a cleavage enzyme for 2 missed cleavage sites. The mass tolerance of precursor ions was set to 40 ppm for the first search and 40 ppm for the main search, and the mass tolerance of fragment ions was set to 0.04 Da. The false-discovery rate (FDR) should be adjusted to <1%. The data in brief had been summarized in Supplementary dataset 1.

### Statistical analysis

Statistical analysis was performed in SPSS 25.0 version software (SPSS Inc., Chicago, USA) or GraphPad Prism 9. The random forest classifier was used for proximity relations of categorical immunophenotypes to train the model. The pre-processed data set was split into a training set and a validation set. The 5-fold training cross-validation was used to prevent overfitting. The receiver operating characteristic (ROC) curve was conducted to assess the random forest classifier with comparing the area under the curve (AUC). Correlation analyses were investigated using the *χ*² test. DFS was defined as the time from randomization until any disease relapse or death from any cause and OS as the time from randomization to death irrespective of cause. For univariable and multivariable Cox proportional hazards regression, 95% confidence intervals (CIs) with two-sided *p* values were used based on the Omnibus test. Kaplan–Meier survival analysis was conducted by the log-rank test. All *P-*values < 0.05 were considered as significant differences.

### Reporting summary

Further information on research design is available in the Nature Research Reporting Summary linked to this article.

## Supplementary information

Supplementary files-merged

Supplementary dataset 1

Reporting Summary

## Data Availability

We are willing to share all of the data and the original code of our report in our published paper with the research community. All relevant data are available from the authors. The mass spectrometry proteomics data have been deposited to the ProteomeXchange Consortium via the PRIDE partner repository with the dataset identifier PXD029186. Please use the link: https://www.ebi.ac.uk/pride/. ProteomeXchange accession: PXD029186.
