# Supplementary material for: Spatial immunophenotypes orchestrate prognosis in triple-negative breast cancer with Miller-Payne grade 4 following neoadjuvant chemotherapy
Source: NPJ Breast Cancer. 2023 Jul 12;9:57. doi: 10.1038/s41523-023-00565-8 (PMC10338497; doi:10.1038/s41523-023-00565-8)
Supplement: Supplementary file 1 — Supplementary files-merged [file 41523_2023_565_MOESM1_ESM.pdf]

Supplementary Table 1. Baseline parameters and distribution of stroma TILs in TNBC subgroup of Miller-Payne 4 with ypN0.

	Total		Low (0-10%)		Intermediate (11-59%)		High (≥60%)		
variable	n	%	n	%	n	%	n	%	*P*-value
Total no.	187	100	46	24.6	72	38.5	69	36.9	-
**Age**									
≤40	79	42.2	16	20.3	33	41.8	30	38.0	0.479
>40	108	57.8	30	27.8	39	36.1	39	36.1	
**Menopausal status**									
Post	106	56.7	22	20.8	46	43.4	38	35.8	0.216
Pre/peri	81	43.3	24	29.6	26	32.1	31	38.3	
**Histology**									
Lobular and others	31	16.6	7	22.6	9	29.0	15	48.4	0.324
Ductal	156	83.4	39	25.0	63	40.4	54	34.6	
**Nuclear grade**									
1-2	97	51.9	18	18.6	39	40.2	40	41.2	0.124
3	90	48.1	28	31.1	33	36.7	29	32.2	
**Prechemotherapy tumor size**									
T1	40	21.4	9	22.5	11	27.5	20	50	0.157
T2	98	52.4	21	21.4	44	44.9	33	33.7	
T3	49	26.2	16	32.7	17	34.7	16	32.7	
**Prechemotherapy node status**									
negative	94	50.3	12	12.8	41	43.6	41	43.6	**0.001**
positive	93	49.7	34	36.6	31	33.3	28	30.1	
**Stage (AJCC staging)**									
I	23	12.3	0	0	11	47.8	12	52.2	**<0.001**
II	94	50.3	17	18.1	36	38.3	41	43.6	
III	70	37.4	29	41.4	25	35.7	16	22.9	
**NACT**									

**regimens**									
Anthracycline and taxane-based	125	66.8	32	25.6	42	33.6	51	40.8	0.229
Anthracycline- based	27	14.4	4	14.8	14	51.9	9	33.3	
Other	35	18.7	10	28.6	16	45.7	9	25.7	

*Abbreviations: TILs, tumor-infiltrating lymphocytes; NACT, neoadjuvant chemotherapy.

χ^2 test for trend. The *P*-values with statistical significance are shown in bold

Supplementary Table 2. Univariable and multivariable Cox regression analysis with respect to disease-free survival in all baseline parameters among the TNBC subgroup of Miller-Payne 4 with ypN0.

patients	Number Of patients n=187	Number Of event n=66	Median DFS	95% CI	Actuarial 5-Years Disease -free survival(%)	Univariate analysis		Multivariate analysis	
						HR(95% CI)	*p*	HR(95 %CI)	*p*
Age, years									
<40	79	28	84.2	(72.415-96.055)	64.0	1	0.978	-	
≥40	108	38	83.7	(75.478-91.870)	63.3	1.007(0.617-1.643)		-	
Histology									
Lobular and others	31	13	82.7	(67.162-98.269)	56.5	1	0.521	-	
Ductal	156	53	84.3	(76.269-92.430)	65.1	0.820(0.446-1.506)		-	
Menopausal status									
Post	106	33	88.4	(78.801-97.968)	67.1	1	0.205	-	
Pre/peri	81	33	80.1	(70.680-89.552)	57.2	1.365(0.842-2.211)		-	
Nuclear grade									
1-2	97	24	95.9	(87.888-104.021)	74.4	1	**0.002**	1	**0.005**
3	90	42	74.4	(64.521-84.4	52.1	2.197(1.328-3.636)		2.081(1.252-3.460)	

				03)					
Prechemotherapy node status									
negative	94	28	92.0	(83.327-100.773)	69.1	1	0.094	-	
positive	93	38	79.2	(69.456-88.840)	58.5	1.515(0.929-2.473)		-	
Prechemotherapy tumor size									
T1	40	9	86.4	(72.277-88.707)	74.6	1	-	-	
T2	98	36	83.7	(73.898-93.471)	62.7	1.765(0.849-3.669)	0.128	-	
T3	49	21	78.7	(66.316-91.166)	56.9	2.268(1.037-4.959)	**0.040**	-	
Stage (AJCC staging)									
I	23	5	98.9	(83.232-114.520)	72.3	1	-	-	
II	94	31	88.8	(79.677-97.901)	65.6	1.596(0.619-4.113)	0.333	-	
III	70	30	76.8	(66.024-87.543)	57.0	2.208(0.855-5.702)	0.102	-	
Stroma TILs									
Low (0-10%)	46	21	74.2	(60.117-88.207)	50.8	1	-	1	-
Intermediate (11-59%)	72	27	80.9	(69.745-91.9	59.7	0.791(0.446-1.404)	0.423	0.873(0.490-1.555)	0.645

				86)					
High (≥60%)	69	18	97.1	(88.333-105.825)	72.8	0.460(0.245-0.865)	**0.016**	0.518(0.274-0.979)	**0.043**
NACT regimens									
Anthracycline and taxane-based	125	43	86.3	(77.746-94.854)	65.9	1	-	-	
Anthracycline- based	27	11	59.2	(50.677-67.663)	61.5	1.187(0.611-2.306)	0.614	-	
Others	35	12	74.6	(62.577-86.696)	58.4	1.053(0.554-2.001)	0.874	-	

*Abbreviations: DFS, disease-free survival; TILs, tumor-infiltrating lymphocytes; NACT, neoadjuvant chemotherapy; CI, confidence interval; HR, hazard ratio.

The *P*-values with statistical significance are shown in bold based on Omnibus test.

Supplementary Table 3. Univariable and multivariable Cox regression analysis with respect to overall survival in all baseline parameters among the TNBC subgroup of Miller-Payne 4 with ypN0.

patients	Number Of patients n=187	Number Of event n=54	Median OS	95% CI	Actuarial 5-Years overall survival(%)	Univariate analysis		Multivariate analysis	
						HR(95% CI)	*p*	HR(95 %CI)	*p*
Age, years									
<40	79	23	91.2	(82.866-99.588)	80.2	1	0.977	-	
≥40	108	31	88.7	(76.561-100.911)	75.0	0.992(0.578-1.702)		-	
Histology									
Lobular and others	31	11	88.1	(72.880-103.334)	72.1	1	0.475	**-**	
Ductal	156	43	90.3	(82.005-98.531)	81.2	0.786(0.405-1.525)		-	
Menopausal status									
Post	106	27	92.7	(83.019-102.305)	83.6	1	0.261	-	
Pre/peri	81	27	88.0	(78.376-97.666)	76.5	1.356(0.795-2.313)		-	
Nuclear grade									
1-2	97	19	100.1	(92.387-107.813)	86.4	1	**0.003**	1	**0.007**
3	90	35	81.1	(70.843-91.4	70.5	2.314(1.322-4.052)		2.183(1.241-3.840)	

				78)					
Prechemotherapy node status									
negative	94	24	95.2	(86.576-103.860)	83.1	1	0.234	-	
positive	93	30	86.6	(76.752-96.558)	76.6	1.385(0.808-2.371)		-	
Prechemotherapy tumor size									
T1	40	7	91.4	(75.724-92.075)	86.4	1	-	-	
T2	98	28	89.7	(79.780-99.677)	80.3	1.758(0.768-4.027)	0.182	-	
T3	49	19	83.0	(70.360-95.713)	71.2	2.621(1.101-6.237)	**0.029**	-	
Stage (AJCC staging)									
I	23	4	102.7	(88.064-117.264)	82.4	1	-	-	
II	94	26	92.8	(83.694-101.884)	80.7	1.612(0.562-4.620)	0.374	-	
III	70	24	85.0	(73.809-96.245)	71.3	2.119(0.735-6.110)	0.165	-	
Stroma TILs									
Low (0-10%)	46	18	79.7	(65.446-93.946)	63.4	1	-	1	-
Intermediate (11-59%)	72	22	87.6	(75.873-99.2	75.8	0.751(0.401-1.406)	0.371	0.835(0.444-1.570)	0.575

				83)					
High (≥60%)	69	14	101.7	(93.611-109.847)	83.2	0.422(0.209-0.850)	**0.016**	0.475(0.235-0.963)	**0.039**
NACT regimens									
Anthracycline and taxane-based	125	33	93.2	(84.601-101.710)	81.5	1	**-**	-	
Anthracycline- based	27	10	60.3	(51.825-68.761)	78.0	1.382(0.681-2.805)	0.371	-	
Others	35	11	75.9	(63.647-88.246)	74.5	1.240(0.627-2.456)	0.536	-	

*Abbreviations: OS, overall survival; TILs, tumor-infiltrating lymphocytes; NACT, neoadjuvant chemotherapy; CI, confidence interval; HR, hazard ratio.

The *P*-values with statistical significance are shown in bold based on Omnibus test.

Supplementary Table 4

MIBI-TOF: Antibodies and channels

Panel (overnight stain)

Antibody target	Provider	Catalog	Clone	Mass channel	Isotope	Titer (µg/mL)
α-SMA	ebioscience	14-9760-82	1A4	141	Pr	0.01
CD19	Fluidigm	3142014D	6OMP31	142	Nd	0.01
CD14	Fluidigm	3144025D	EPR3653	144	Nd	0.01
CD33	Fluidigm	3145017D	Polyclonal	145	Nd	0.01
CD16	Fluidigm	3146020D	EPR16784	146	Nd	0.01
Vimentin	Abcam	8978	RV202	148	Nd	0.01
CD11b/Mac-1	Fluidigm	3149028D	EPR1344	149	Sm	0.01
CD274/PD-L1	Fluidigm	3150031D	E1L3N	150	Nd	0.01
TGF-β	Biolegend	846802	092B5	151	Eu	0.01
CD56	Abcam	9018	RNL-1	152	Sm	0.01
CD15	Biolegend	301902	HI98	153	Eu	0.01
CD11c	Fluidigm	3154025D	Polyclonal	154	Sm	0.01
Foxp3	Biolegend	320102	206D	155	Gd	0.01
CD4	Fluidigm	3156033D	EPR6855	156	Gd	0.01
CD324/E-Cadherin	Abcam	231303	4A2	158	Gd	0.01
CD68	Biolegend	916104	KP1	159	Tb	0.01
Tim-3	ebioscience	14-5871-82	8B.2C12	160	Gd	0.01
CD8a	Fluidigm	3162035D	D8A8Y	162	Dy	0.01
CD279/PD-1	Fluidigm	3165039D	EPR4877(2)	165	Ho	0.01
CD45	Biolegend	368502	2D1	166	Er	0.01
Bcl-6	Abcam	243920	SP155	167	Er	0.01
Ki-67	Biolegend	350502	Ki-67	168	Er	0.01
collagen I	Abcam	88147	3G3	169	Tm	0.01
CD3	Fluidigm	3170019D	Polyclonal, C-Termina	170	Er	0.01
HLA-DR	Fluidigm	3174023D	YE2/36 HLK	174	Yb	0.01
T-bet	Invitrogen	700059	16H4L5	175	Lu	0.01
GATA-3	Abcam	ab214804	EPR16651	176	Yb	0.01
IL-10	Abcam	ab134742	4A7-25-17	171	Yb	0.01
IFN-γ	Abcam	9657	Polyclonal	147	Sm	0.01

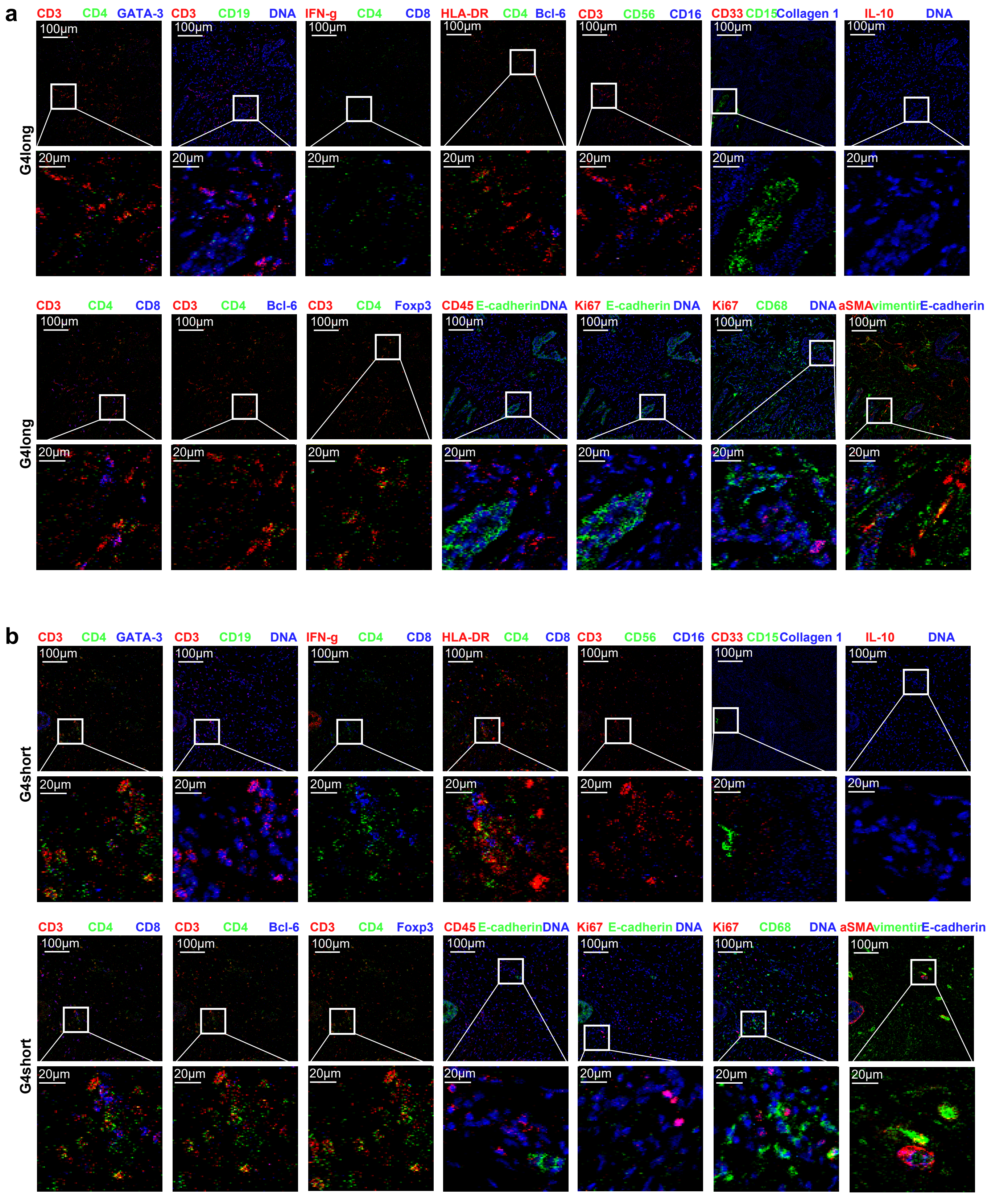
a
G4long
CD3 CD4 GATA-3
CD3 CD19 DNA
IFN-g CD4 CD8
HLA-DR CD4 Bcl-6
CD3 CD56 CD16
CD33 CD15 Collagen 1
IL-10 DNA
CD3 CD4 CD8
CD3 CD4 Bcl-6
CD3 CD4 Foxp3
CD45 E-cadherin DNA
Ki67 E-cadherin DNA
Ki67 CD68 DNA
aSMA vimentin E-cadherin
100μm
20μm
b
G4short
CD3 CD4 GATA-3
CD3 CD19 DNA
IFN-g CD4 CD8
HLA-DR CD4 CD8
CD3 CD56 CD16
CD33 CD15 Collagen 1
IL-10 DNA
CD3 CD4 CD8
CD3 CD4 Bcl-6
CD3 CD4 Foxp3
CD45 E-cadherin DNA
Ki67 E-cadherin DNA
Ki67 CD68 DNA
aSMA vimentin E-cadherin

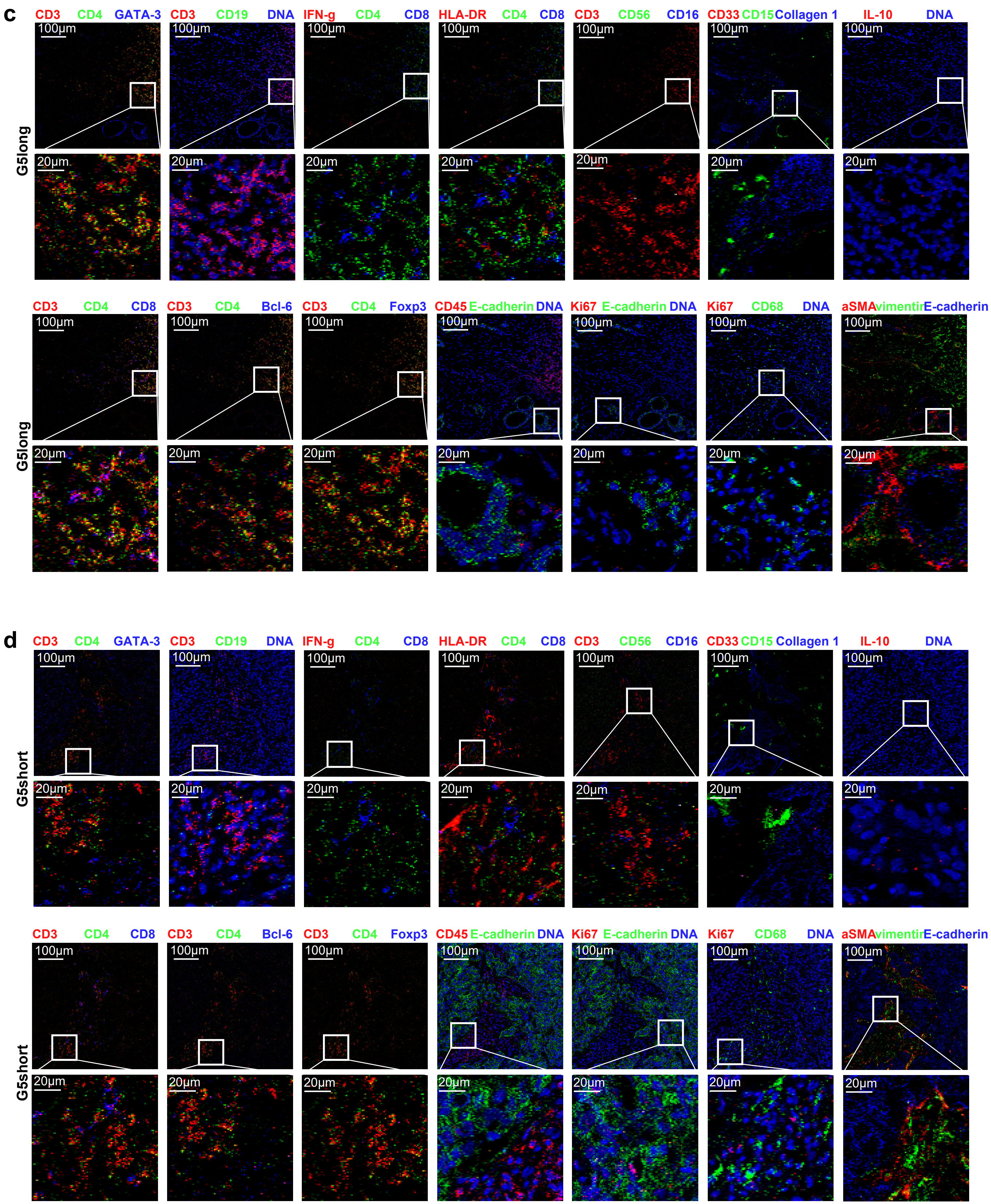

Supplementary Figure 1. The other different spatial immunophenotypes in Miller-Payne 4/5 groups associated with good and poor prognosis. The other different spatial immunophenotypes in Miller-Payne 4/5 groups associated with good and poor prognosis. Upward side, 500μm× 500μm region, scale bar: 100μm; Downward side, 100μm× 100μm, scale bar: 20μm. G4=Miller-Payne 4; G5=Miller-Payne 5.

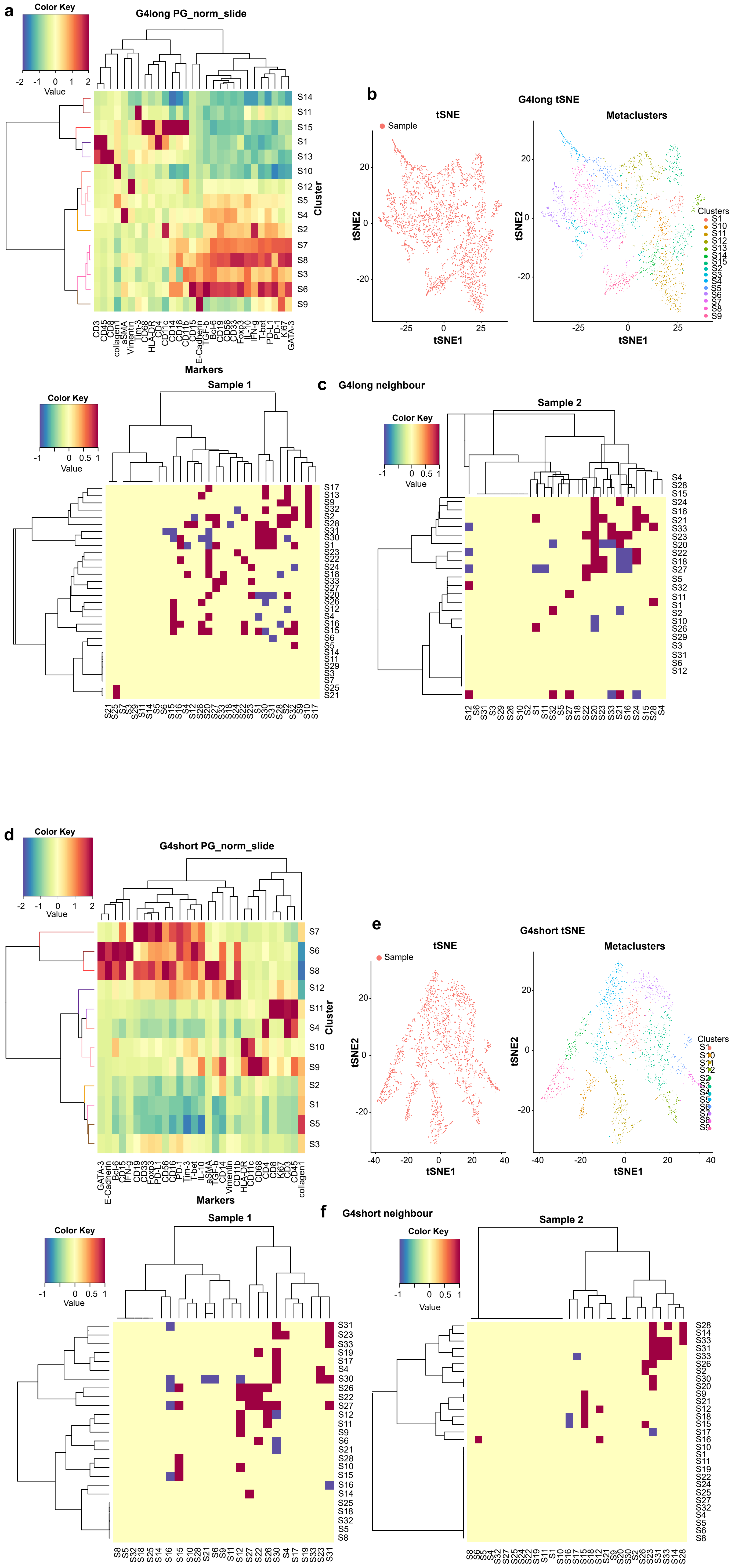

a
G4long PG_norm_slide
Color Key
Value
Cluster
Markers
b
G4long tSNE
tSNE
Sample
Metaclusters
Clusters
tSNE1
tSNE2
c
G4long neighbour
Sample 1
Sample 2
d
G4short PG_norm_slide
e
G4short tSNE
f
G4short neighbour

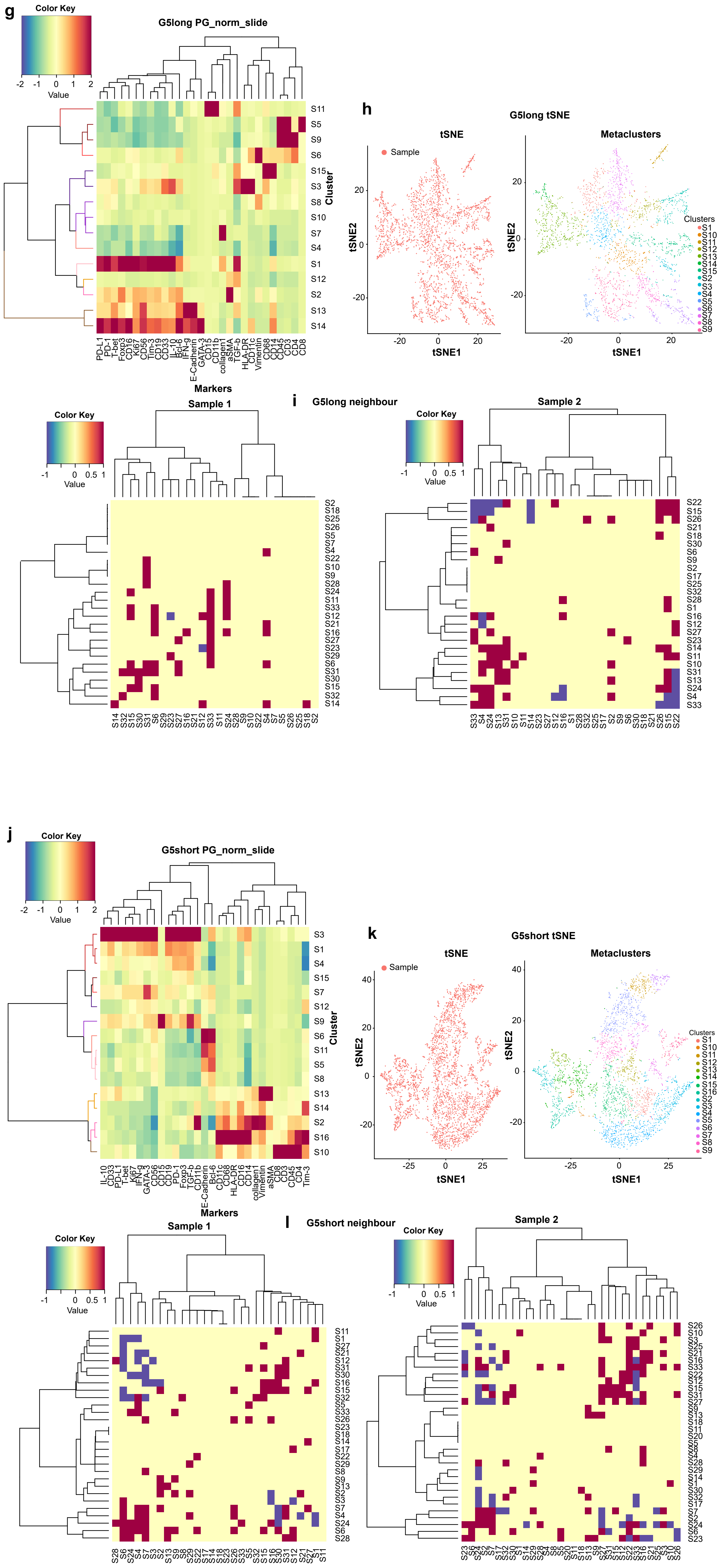

Supplementary Figure 2. Separated analysis of spatial distribution of immunophenotypes in Miller-Payne 4/5 patients with good and poor prognosis respectively. (a, d, g, j) Immunophenotypes were standardly clustered by protein expression markers in each group. Scale from -2 to 2. (b, e, h, k) tSNE plots showed the similarity of immunophenotypes (left), and divided immunophenotypes populations according to clusters (right). (c, f, i, l) Heatmaps denote spatial proximity z scores between pairs of clusters (y axis) in regions of all neighbours in two biological duplicate samples, scaled from -1 to 1. TIL=tumor-infiltrating lymphocytes. t-SNE, t-Distributed Stochastic Neighbor Embedding. G4=Miller-Payne 4; G5=Miller-Payne 5.

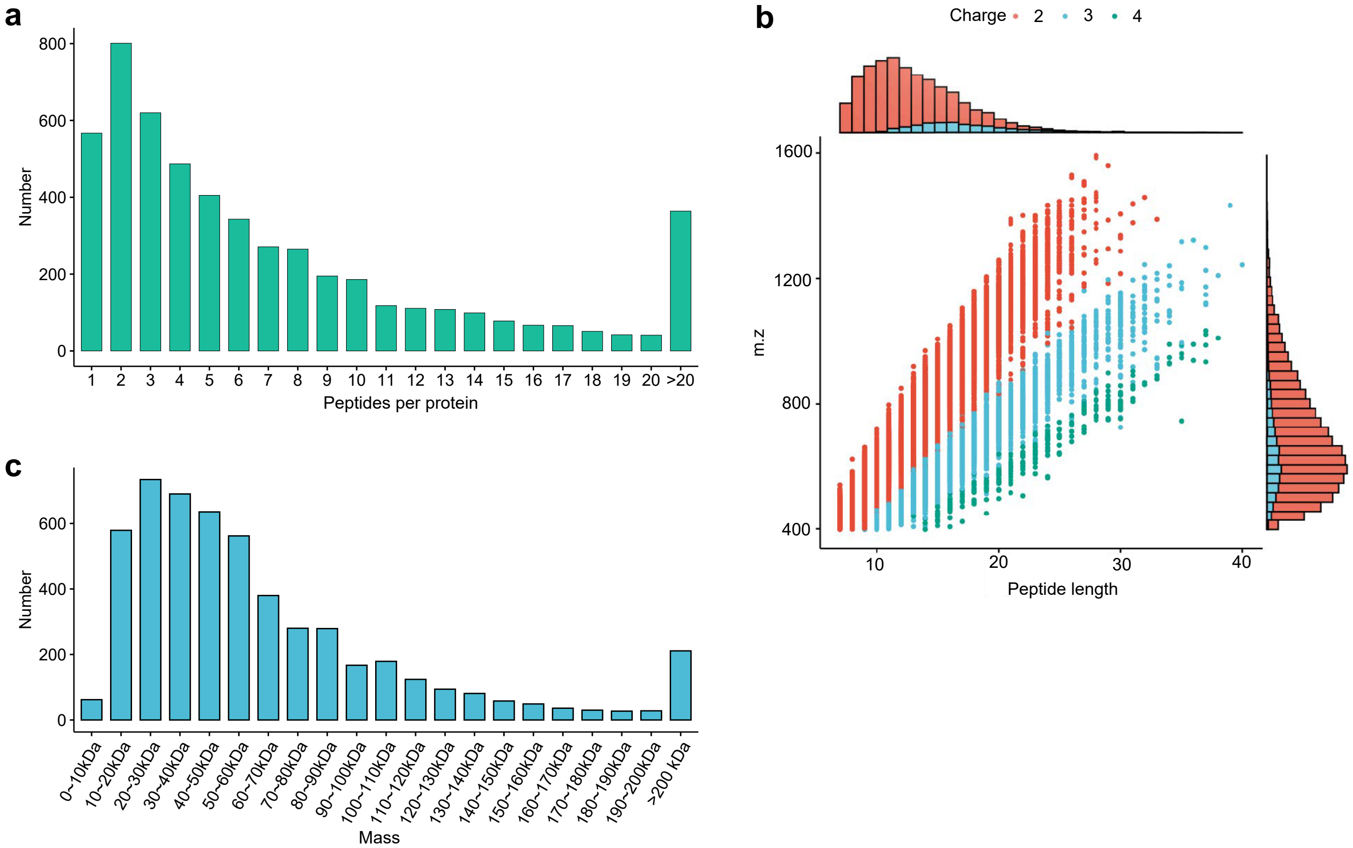

Supplementray Figure 3. Data quality control of identified proteins. (a) Protein coverage distribution; (b) Peptide length distribution, mainly range 7-20; (c) Molecular weight distribution of protein. Units: thousand daltons, kDa.

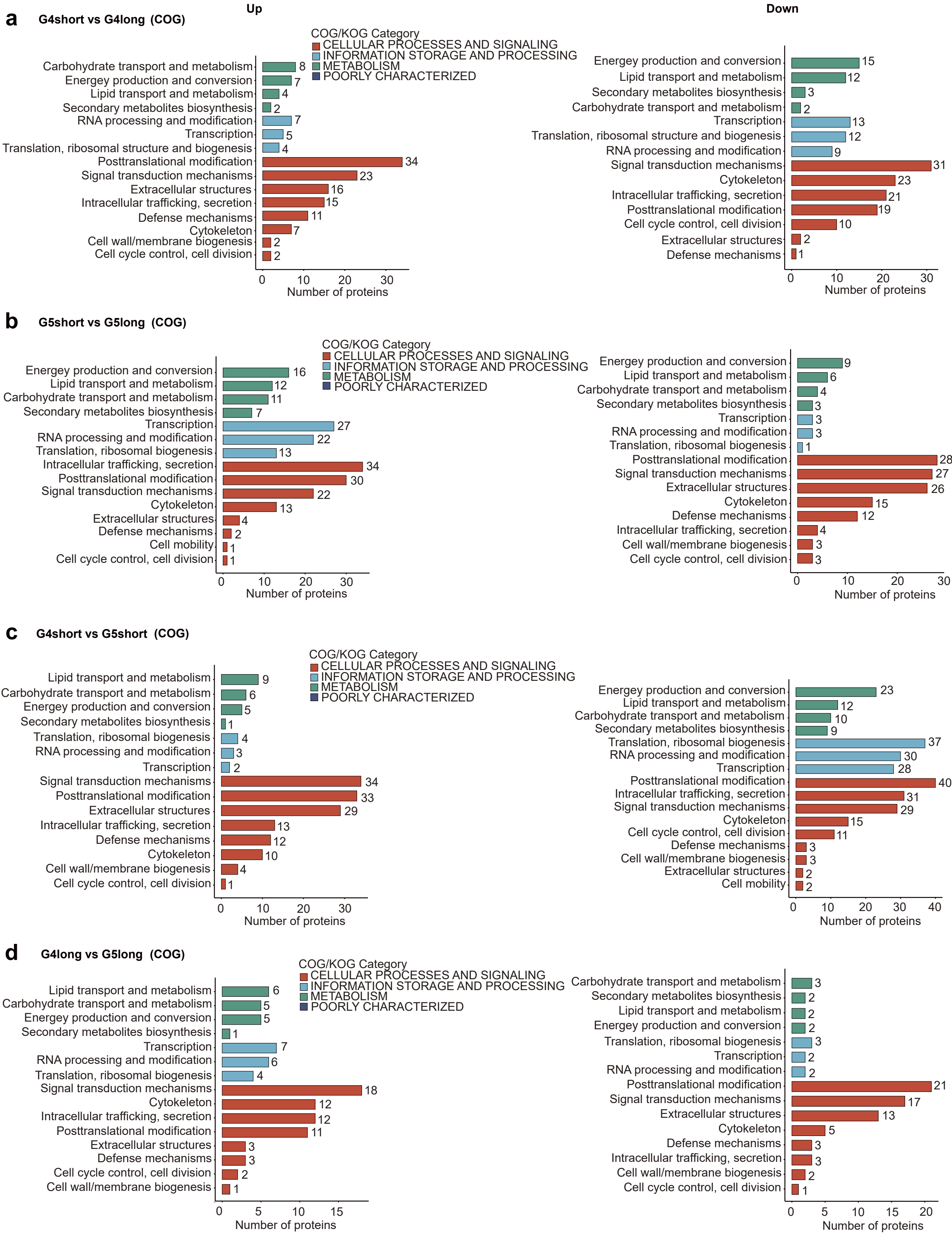

Supplementary Figure 4. Clusters of Orthologous Groups of proteins (COG) annotation of differentially expressed proteins related to immune processing based on CV values (top 10 results), in (a) for G4short vs G4long, (b) for G5short vs G5long, (c) for G4short vs G5short and (d) for G4long vs G5long group. CV, coefficient of variation. G4=Miller-Payne 4; G5=Miller-Payne 5.

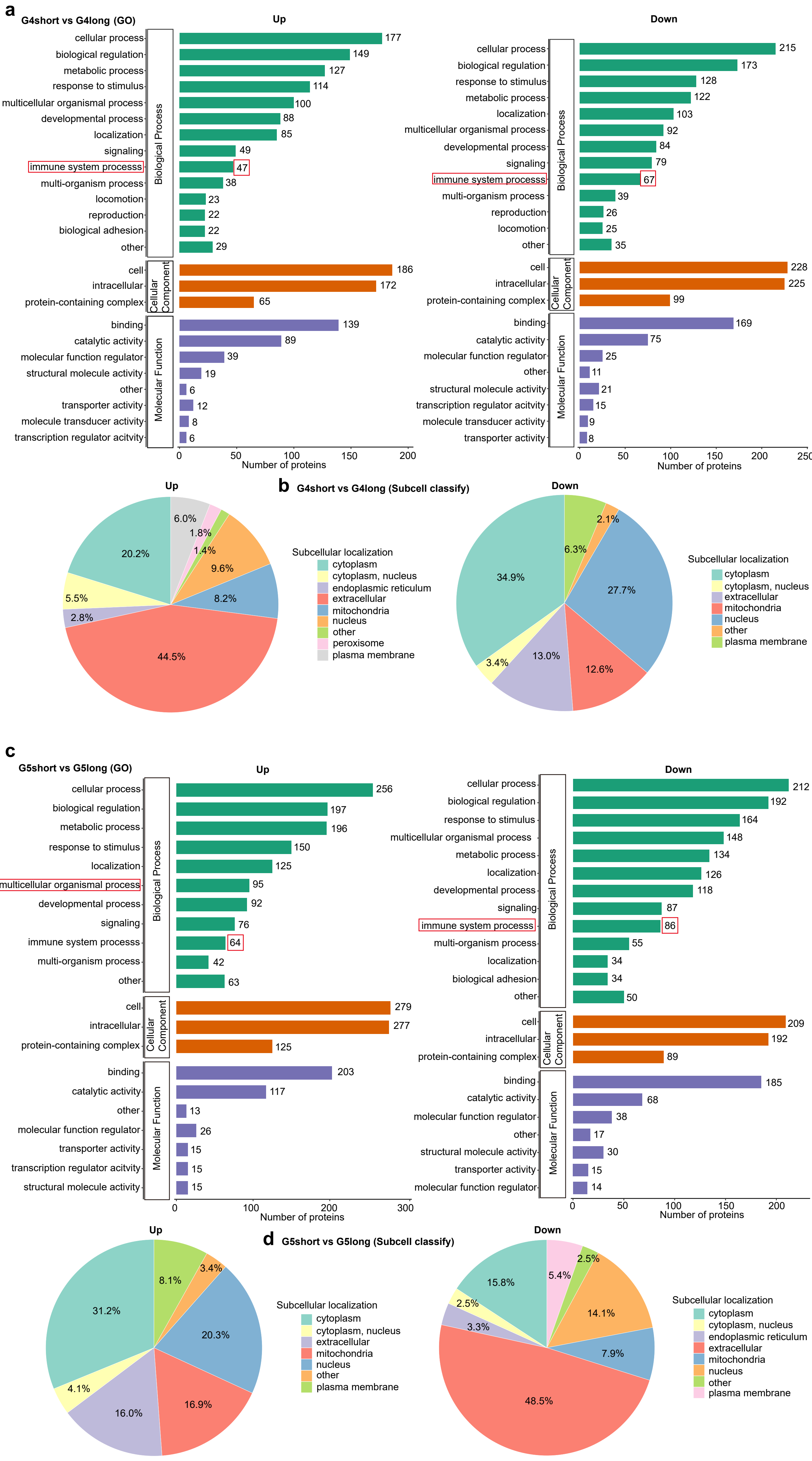
a
G4short vs G4long (GO)
Up
cellular process 177
biological regulation 149
metabolic process 127
response to stimulus 114
multicellular organismal process 100
developmental process 88
localization 85
signaling 49
immune system processs 47
multi-organism process 38
locomotion 23
reproduction 22
biological adhesion 22
other 29
Biological Process
cell 186
intracellular 172
protein-containing complex 65
Cellular Component
binding 139
catalytic activity 89
molecular function regulator 39
structural molecule activity 19
other 6
transporter activity 12
molecule transducer activity 8
transcription regulator acitvity 6
Molecular Function
0 50 100 150 200
Number of proteins
Down
cellular process 215
biological regulation 173
response to stimulus 128
metabolic process 122
localization 103
multicellular organismal process 92
developmental process 84
signaling 79
immune system processs 67
multi-organism process 39
reproduction 26
locomotion 25
other 35
Biological Process
cell 228
intracellular 225
protein-containing complex 99
Cellular Component
binding 169
catalytic activity 75
molecular function regulator 25
other 11
structural molecule activity 21
transcription regulator acitvity 15
molecule transducer activity 9
transporter activity 8
Molecular Function
0 50 100 150 200 250
Number of proteins
b
G4short vs G4long (Subcell classify)
Up
20.2%
6.0%
1.8%
1.4%
9.6%
8.2%
5.5%
2.8%
44.5%
Subcellular localization
cytoplasm
cytoplasm, nucleus
endoplasmic reticulum
extracellular
mitochondria
nucleus
other
peroxisome
plasma membrane
Down
34.9%
6.3%
2.1%
27.7%
3.4%
13.0%
12.6%
Subcellular localization
cytoplasm
cytoplasm, nucleus
extracellular
mitochondria
nucleus
other
plasma membrane
c
G5short vs G5long (GO)
Up
cellular process 256
biological regulation 197
metabolic process 196
response to stimulus 150
localization 125
multicellular organismal process 95
developmental process 92
signaling 76
immune system processs 64
multi-organism process 42
other 63
Biological Process
cell 279
intracellular 277
protein-containing complex 125
Cellular Component
binding 203
catalytic activity 117
other 13
molecular function regulator 26
transporter activity 15
transcription regulator acitvity 15
structural molecule activity 15
Molecular Function
0 100 200 300
Number of proteins
Down
cellular process 212
biological regulation 192
response to stimulus 164
multicellular organismal process 148
metabolic process 134
localization 126
developmental process 118
signaling 87
immune system processs 86
multi-organism process 55
localization 34
biological adhesion 34
other 50
Biological Process
cell 209
intracellular 192
protein-containing complex 89
Cellular Component
binding 185
catalytic activity 68
molecular function regulator 38
other 17
structural molecule activity 30
transporter activity 15
molecular function regulator 14
Molecular Function
0 50 100 150 200
Number of proteins
d
G5short vs G5long (Subcell classify)
Up
31.2%
8.1%
3.4%
20.3%
4.1%
16.0%
16.9%
Subcellular localization
cytoplasm
cytoplasm, nucleus
extracellular
mitochondria
nucleus
other
plasma membrane
Down
15.8%
5.4%
2.5%
14.1%
2.5%
3.3%
7.9%
48.5%
Subcellular localization
cytoplasm
cytoplasm, nucleus
endoplasmic reticulum
extracellular
mitochondria
nucleus
other
plasma membrane

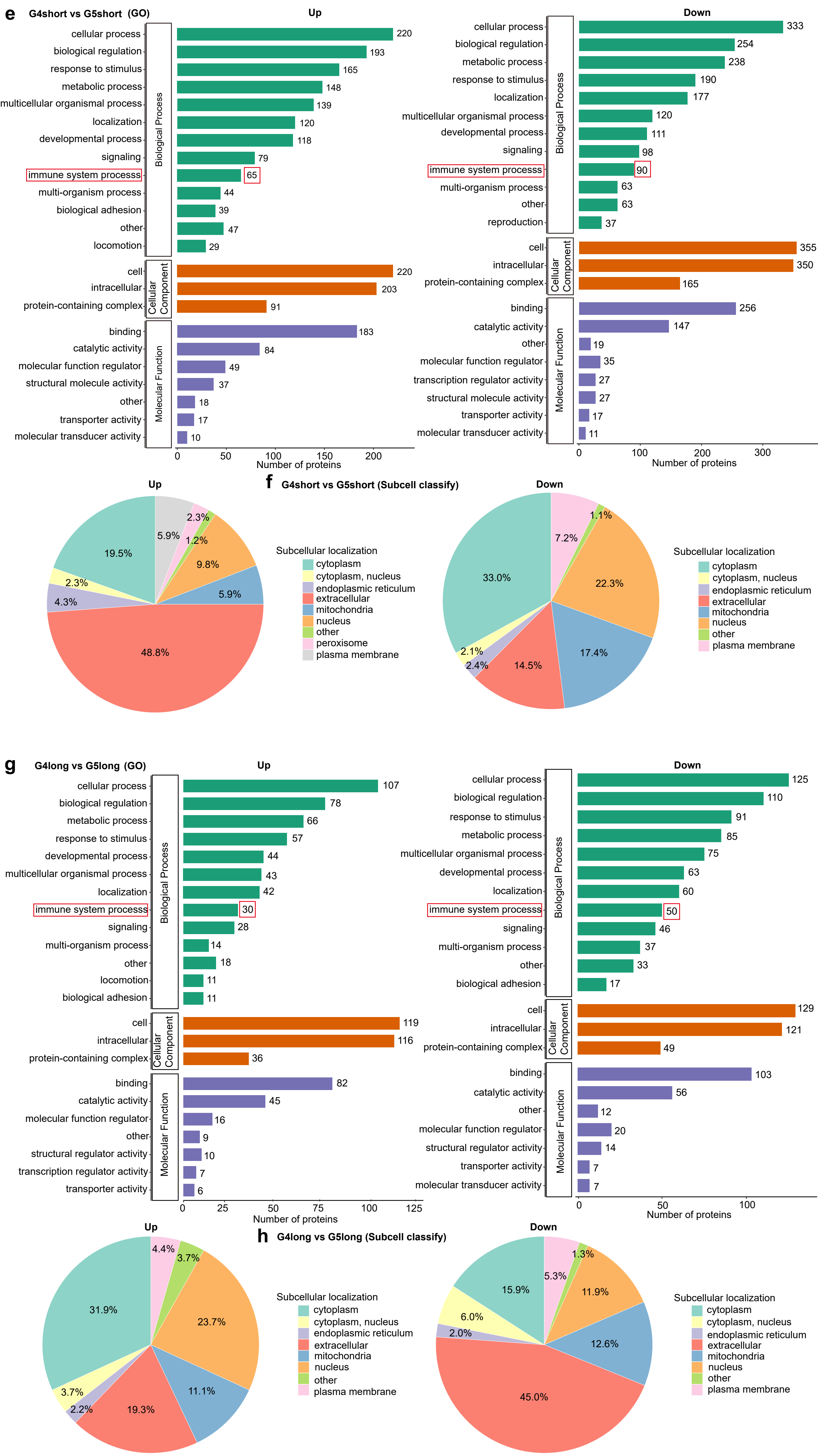

Supplementary Figure 5. GeneOntology (GO) annotation (top 10 results), and Wolfpsort-based classification of subcellular localization based on CV values. (a, b) for G4short vs G4long, (c, d) for G5short vs G5long, (e, f) for G4short vs G5short and (g, h) for G4long vs G5long group. Immune system process was significantly marked. CV, coefficient of variation. G4=Miller-Payne 4; G5=Miller-Payne 5.

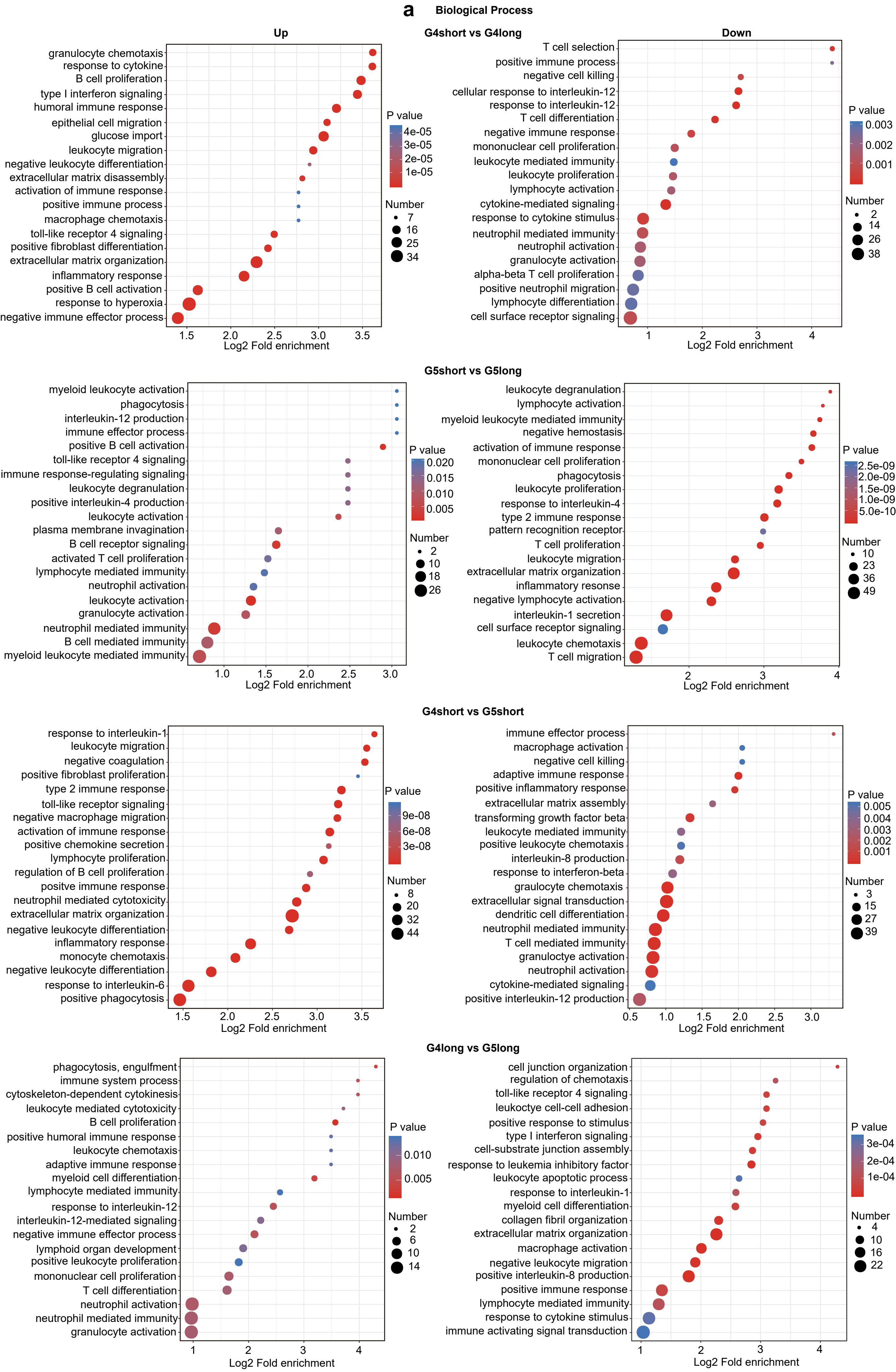

a
Biological Process
G4short vs G4long
Up
granulocyte chemotaxis
response to cytokine
B cell proliferation
type I interferon signaling
humoral immune response
epithelial cell migration
glucose import
leukocyte migration
negative leukocyte differentiation
extracellular matrix disassembly
activation of immune response
positive immune process
macrophage chemotaxis
toll-like receptor 4 signaling
positive fibroblast differentiation
extracellular matrix organization
inflammatory response
positive B cell activation
response to hyperoxia
negative immune effector process
1.5
2.0
2.5
3.0
3.5
Log2 Fold enrichment
P value
4e-05
3e-05
2e-05
1e-05
Number
7
16
25
34
Down
T cell selection
positive immune process
negative cell killing
cellular response to interleukin-12
response to interleukin-12
T cell differentiation
negative immune response
mononuclear cell proliferation
leukocyte mediated immunity
leukocyte proliferation
lymphocyte activation
cytokine-mediated signaling
response to cytokine stimulus
neutrophil mediated immunity
neutrophil activation
granulocyte activation
alpha-beta T cell proliferation
positive neutrophil migration
lymphocyte differentiation
cell surface receptor signaling
1
2
3
4
Log2 Fold enrichment
P value
0.003
0.002
0.001
Number
2
14
26
38
G5short vs G5long
myeloid leukocyte activation
phagocytosis
interleukin-12 production
immune effector process
positive B cell activation
toll-like receptor 4 signaling
immune response-regulating signaling
leukocyte degranulation
positive interleukin-4 production
leukocyte activation
plasma membrane invagination
B cell receptor signaling
activated T cell proliferation
lymphocyte mediated immunity
neutrophil activation
leukocyte activation
granulocyte activation
neutrophil mediated immunity
B cell mediated immunity
myeloid leukocyte mediated immunity
1.0
1.5
2.0
2.5
3.0
Log2 Fold enrichment
P value
0.020
0.015
0.010
0.005
Number
2
10
18
26
leukocyte degranulation
lymphocyte activation
myeloid leukocyte mediated immunity
negative hemostasis
activation of immune response
mononuclear cell proliferation
phagocytosis
leukocyte proliferation
response to interleukin-4
type 2 immune response
pattern recognition receptor
T cell proliferation
leukocyte migration
extracellular matrix organization
inflammatory resonse
negative lymphocyte activation
interleukin-1 secretion
cell surface receptor signaling
leukocyte chemotaxis
T cell migration
2
3
4
Log2 Fold enrichment
P value
2.5e-09
2.0e-09
1.5e-09
1.0e-09
5.0e-10
Number
10
23
36
49
G4short vs G5short
response to interleukin-1
leukocyte migration
negative coagulation
positive fibroblast proliferation
type 2 immune response
toll-like receptor signaling
negative macrophage migration
activation of immune response
positive chemokine secretion
lymphocyte proliferation
regulation of B cell proliferation
positve immune response
neutrophil mediated cytotoxicity
extracellular matrix organization
negative leukocyte differentiation
inflammatory response
monocyte chemotaxis
negative leukocyte differentiation
response to interleukin-6
positive phagocytosis
1.5
2.0
2.5
3.0
3.5
Log2 Fold enrichment
P value
9e-08
6e-08
3e-08
Number
8
20
32
44
immune effector process
macrophage activation
negative cell killing
adaptive immune response
positive inflammatory response
extracellular matrix assembly
transforming growth factor beta
leukocyte mediated immunity
positive leukocyte chemotaxis
interleukin-8 production
response to interferon-beta
graulocyte chemotaxis
extracellular signal transduction
dendritic cell differentiation
neutrophil mediated immunity
T cell mediated immunity
granulocyte activation
neutrophil activation
cytokine-mediated signaling
positive interleukin-12 production
0.5
1.0
1.5
2.0
2.5
3.0
Log2 Fold enrichment
P value
0.005
0.004
0.003
0.002
0.001
Number
3
15
27
39
G4long vs G5long
phagocytosis, engulfment
immune system process
cytoskeleton-dependent cytokinesis
leukocyte mediated cytotoxicity
B cell proliferation
positive humoral immune response
leukocyte chemotaxis
adaptive immune response
myeloid cell differentiation
lymphocyte mediated immunity
response to interleukin-12
interleukin-12-mediated signaling
negative immune effector process
lymphoid organ development
positive leukocyte proliferation
mononuclear cell proliferation
T cell differentiation
neutrophil activation
neutrophil mediated immunity
granulocyte activation
1
2
3
4
Log2 Fold enrichment
P value
0.010
0.005
Number
2
6
10
14
cell junction organization
regulation of chemotaxis
toll-like receptor 4 signaling
leukoctye cell-cell adhesion
positive response to stimulus
type I interferon signaling
cell-substrate junction assembly
response to leukemia inhibitory factor
leukocyte apoptotic process
response to interleukin-1
myeloid cell differentiation
collagen fibril organization
extracellular matrix organization
macrophage activation
negative leukocyte migration
positive interleukin-8 production
positive immune response
lymphocyte mediated immunity
response to cytokine stimulus
immune activating signal transduction
1
2
3
4
Log2 Fold enrichment
P value
3e-04
2e-04
1e-04
Number
4
10
16
22

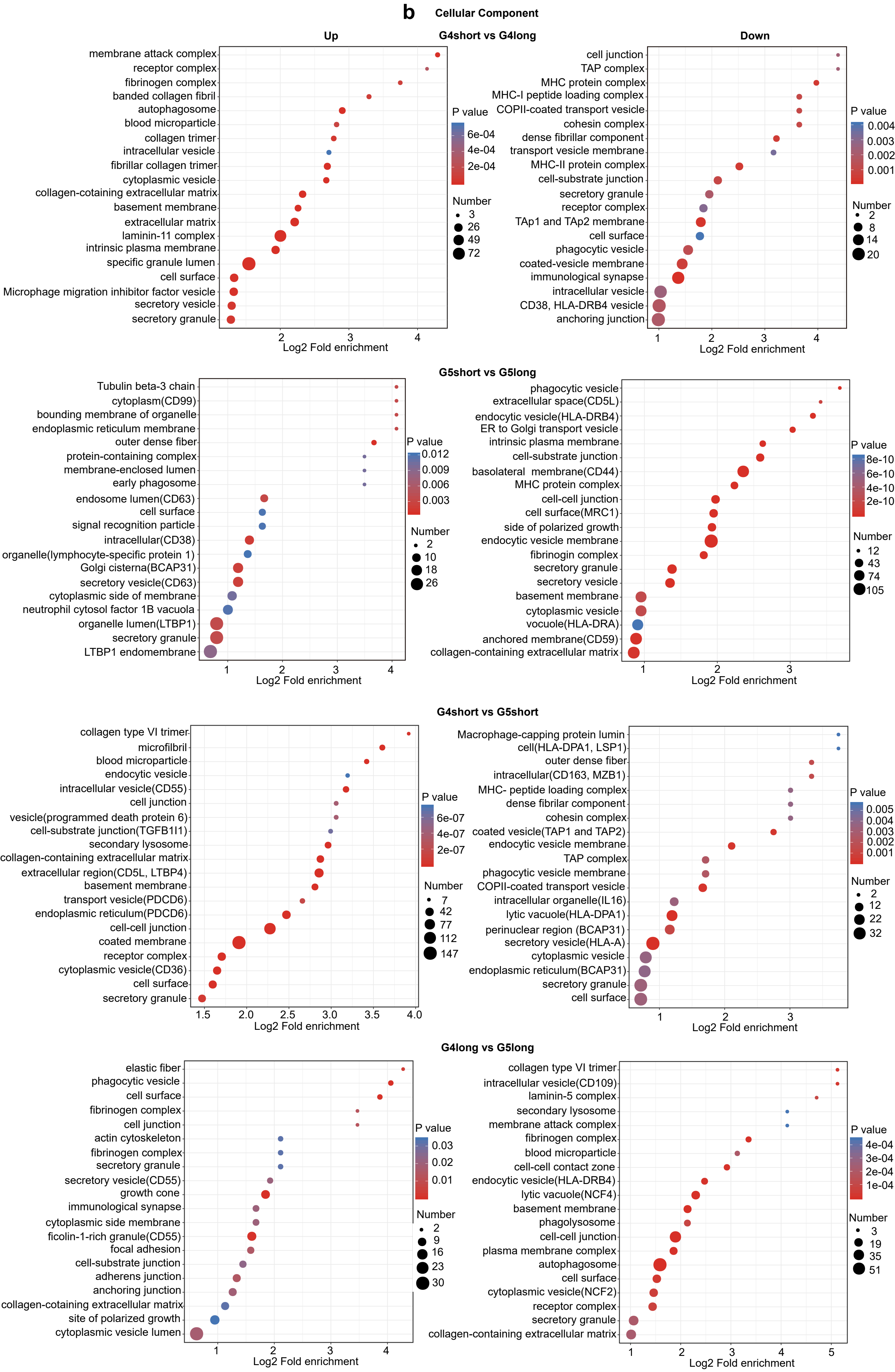

b
Cellular Component
G4short vs G4long
Up
membrane attack complex
receptor complex
fibrinogen complex
banded collagen fibril
autophagosome
blood microparticle
collagen trimer
intracellular vesicle
fibrillar collagen trimer
cytoplasmic vesicle
collagen-cotaining extracellular matrix
basement membrane
extracellular matrix
laminin-11 complex
intrinsic plasma membrane
specific granule lumen
cell surface
Microphage migration inhibitor factor vesicle
secretory vesicle
secretory granule
Log2 Fold enrichment
P value
6e-04
4e-04
2e-04
Number
3
26
49
72
Down
cell junction
TAP complex
MHC protein complex
MHC-I peptide loading complex
COPII-coated transport vesicle
cohesin complex
dense fibrillar component
transport vesicle membrane
MHC-II protein complex
cell-substrate junction
secretory granule
receptor complex
TAp1 and TAp2 membrane
cell surface
phagocytic vesicle
coated-vesicle membrane
immunological synapse
intracellular vesicle
CD38, HLA-DRB4 vesicle
anchoring junction
Log2 Fold enrichment
P value
0.004
0.003
0.002
0.001
Number
2
8
14
20
G5short vs G5long
Tubulin beta-3 chain
cytoplasm(CD99)
bounding membrane of organelle
endoplasmic reticulum membrane
outer dense fiber
protein-containing complex
membrane-enclosed lumen
early phagosome
endosome lumen(CD63)
cell surface
signal recognition particle
intracellular(CD38)
organelle(lymphocyte-specific protein 1)
Golgi cisterna(BCAP31)
secretory vesicle(CD63)
cytoplasmic side of membrane
neutrophil cytosol factor 1B vacuola
organelle lumen(LTBP1)
secretory granule
LTBP1 endomembrane
Log2 Fold enrichment
P value
0.012
0.009
0.006
0.003
Number
2
10
18
26
phagocytic vesicle
extracellular space(CD5L)
endocytic vesicle(HLA-DRB4)
ER to Golgi transport vesicle
intrinsic plasma membrane
cell-substrate junction
basolateral membrane(CD44)
MHC protein complex
cell-cell junction
cell surface(MRC1)
side of polarized growth
endocytic vesicle membrane
fibrinogin complex
secretory granule
secretory vesicle
basement membrane
cytoplasmic vesicle
vocuole(HLA-DRA)
anchored membrane(CD59)
collagen-containing extracellular matrix
Log2 Fold enrichment
P value
8e-10
6e-10
4e-10
2e-10
Number
12
43
74
105
G4short vs G5short
collagen type VI trimer
microfilbril
blood microparticle
endocytic vesicle
intracellular vesicle(CD55)
cell junction
vesicle(programmed death protein 6)
cell-substrate junction(TGFB1I1)
secondary lysosome
collagen-containing extracellular matrix
extracellular region(CD5L, LTBP4)
basement membrane
transport vesicle(PDCD6)
endoplasmic reticulum(PDCD6)
cell-cell junction
coated membrane
receptor complex
cytoplasmic vesicle(CD36)
cell surface
secretory granule
Log2 Fold enrichment
P value
6e-07
4e-07
2e-07
Number
7
42
77
112
147
Macrophage-capping protein lumin
cell(HLA-DPA1, LSP1)
outer dense fiber
intracellular(CD163, MZB1)
MHC- peptide loading complex
dense fibrilar component
cohesin complex
coated vesicle(TAP1 and TAP2)
endocytic vesicle membrane
TAP complex
phagocytic vesicle membrane
COPII-coated transport vesicle
intracellular organelle(IL16)
lytic vacuole(HLA-DPA1)
perinuclear region (BCAP31)
secretory vesicle(HLA-A)
cytoplasmic vesicle
endoplasmic reticulum(BCAP31)
secretory granule
cell surface
Log2 Fold enrichment
P value
0.005
0.004
0.003
0.002
0.001
Number
2
12
22
32
G4long vs G5long
elastic fiber
phagocytic vesicle
cell surface
fibrinogen complex
cell junction
actin cytoskeleton
fibrinogen complex
secretory granule
secretory vesicle(CD55)
growth cone
immunological synapse
cytoplasmic side membrane
ficolin-1-rich granule(CD55)
focal adhesion
cell-substrate junction
adherens junction
anchoring junction
collagen-cotaining extracellular matrix
site of polarized growth
cytoplasmic vesicle lumen
Log2 Fold enrichment
P value
0.03
0.02
0.01
Number
2
9
16
23
30
collagen type VI trimer
intracellular vesicle(CD109)
laminin-5 complex
secondary lysosome
membrane attack complex
fibrinogen complex
blood microparticle
cell-cell contact zone
endocytic vesicle(HLA-DRB4)
lytic vacuole(NCF4)
basement membrane
phagolysosome
cell-cell junction
plasma membrane complex
autophagosome
cell surface
cytoplasmic vesicle(NCF2)
receptor complex
secretory granule
collagen-containing extracellular matrix
Log2 Fold enrichment
P value
4e-04
3e-04
2e-04
1e-04
Number
3
19
35
51

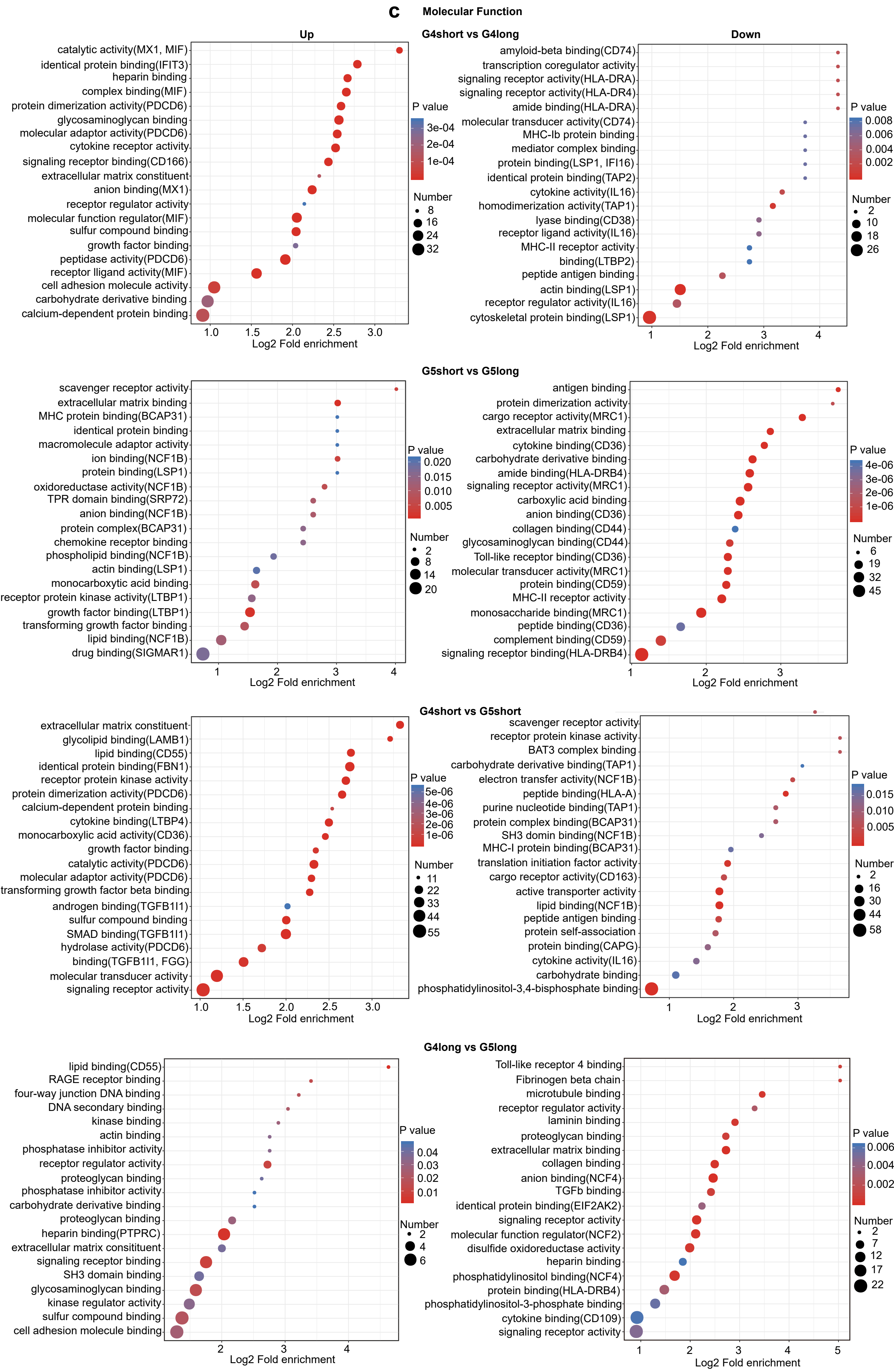

Supplementary Figure 6. GO enrichment-based clustering analysis of the differentially expressed proteins for (a) Biological processes, (b) Cellular components and (c) Molecular function related to immune response in G4short vs G4long, G5short vs G5long, G4short vs G5short and G4long vs G5long group. Red represents a strong degree of enrichment, and blue represents a weak degree of enrichment. The size of point indicates the number of enriched proteins. G4=Miller-Payne 4; G5=Miller-Payne 5.

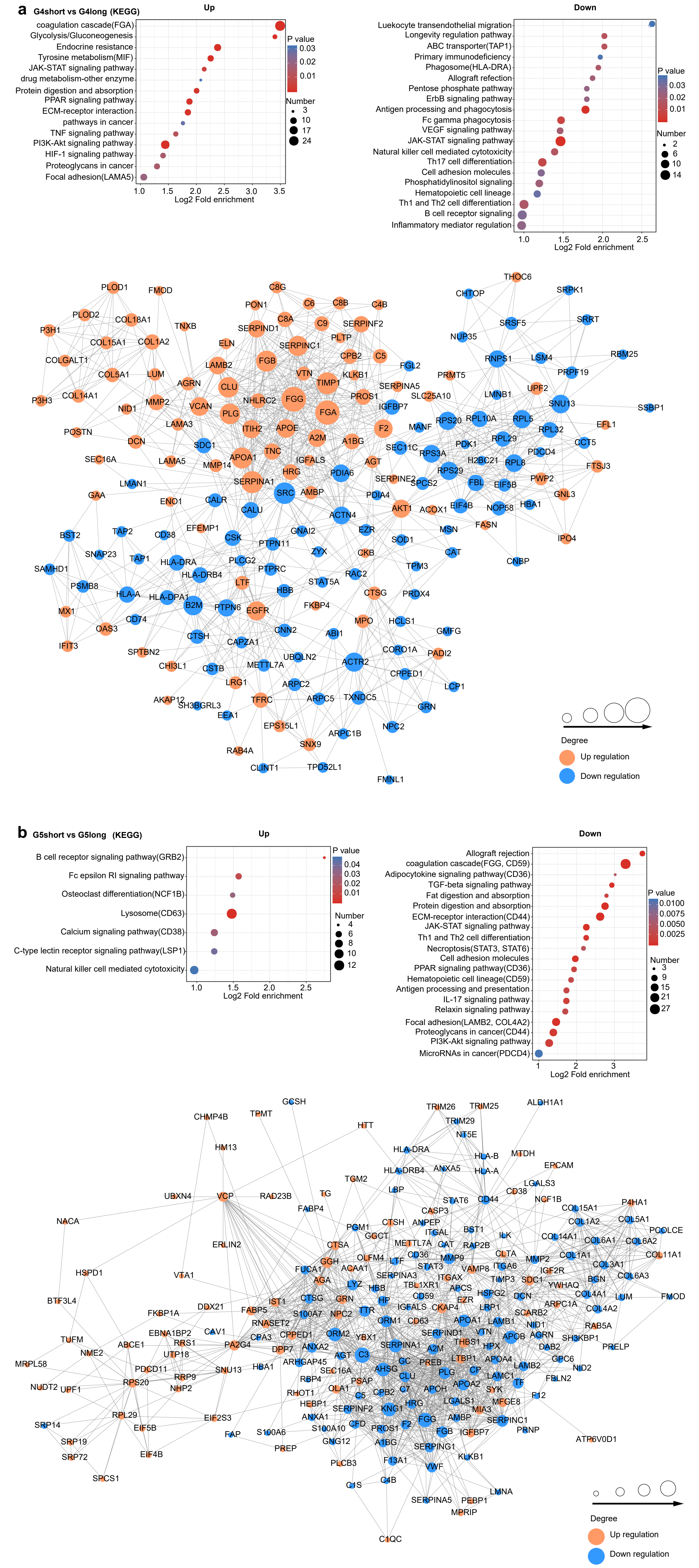
a
G4short vs G4long (KEGG)
Up
coagulation cascade(FGA)
Glycolysis/Gluconeogenesis
Endocrine resistance
Tyrosine metabolism(MIF)
JAK-STAT signaling pathway
drug metabolism-other enzyme
Protein digestion and absorption
PPAR signaling pathway
ECM-receptor interaction
pathways in cancer
TNF signaling pathway
PI3K-Akt signaling pathway
HIF-1 signaling pathway
Proteoglycans in cancer
Focal adhesion(LAMA5)
Log2 Fold enrichment
P value
Number
Down
Luekocyte transendothelial migration
Longevity regulation pathway
ABC transporter(TAP1)
Primary immunodeficiency
Phagosome(HLA-DRA)
Allograft refection
Pentose phosphate pathway
ErbB signaling pathway
Antigen processing and phagocytosis
Fc gamma phagocytosis
VEGF signaling pathway
JAK-STAT signaling pathway
Natural killer cell mediated cytotoxicity
Th17 cell differentiation
Cell adhesion molecules
Phosphatidylinositol signaling
Hematopoietic cell lineage
Th1 and Th2 cell differentiation
B cell receptor signaling
Inflammatory mediator regulation
Log2 Fold enrichment
Degree
Up regulation
Down regulation
b
G5short vs G5long (KEGG)
Up
B cell receptor signaling pathway(GRB2)
Fc epsilon RI signaling pathway
Osteoclast differentiation(NCF1B)
Lysosome(CD63)
Calcium signaling pathway(CD38)
C-type lectin receptor signaling pathway(LSP1)
Natural killer cell mediated cytotoxicity
Log2 Fold enrichment
P value
Number
Down
Allograft rejection
coagulation cascade(FGG, CD59)
Adipocytokine signaling pathway(CD36)
TGF-beta signaling pathway
Fat digestion and absorption
Protein digestion and absorption
ECM-receptor interaction(CD44)
JAK-STAT signaling pathway
Th1 and Th2 cell differentiation
Necroptosis(STAT3, STAT6)
Cell adhesion molecules
PPAR signaling pathway(CD36)
Hematopoietic cell lineage(CD59)
Antigen processing and presentation
IL-17 signaling pathway
Relaxin signaling pathway
Focal adhesion(LAMB2, COL4A2)
Proteoglycans in cancer(CD44)
PI3K-Akt signaling pathway
MicroRNAs in cancer(PDCD4)
Log2 Fold enrichment
Degree
Up regulation
Down regulation

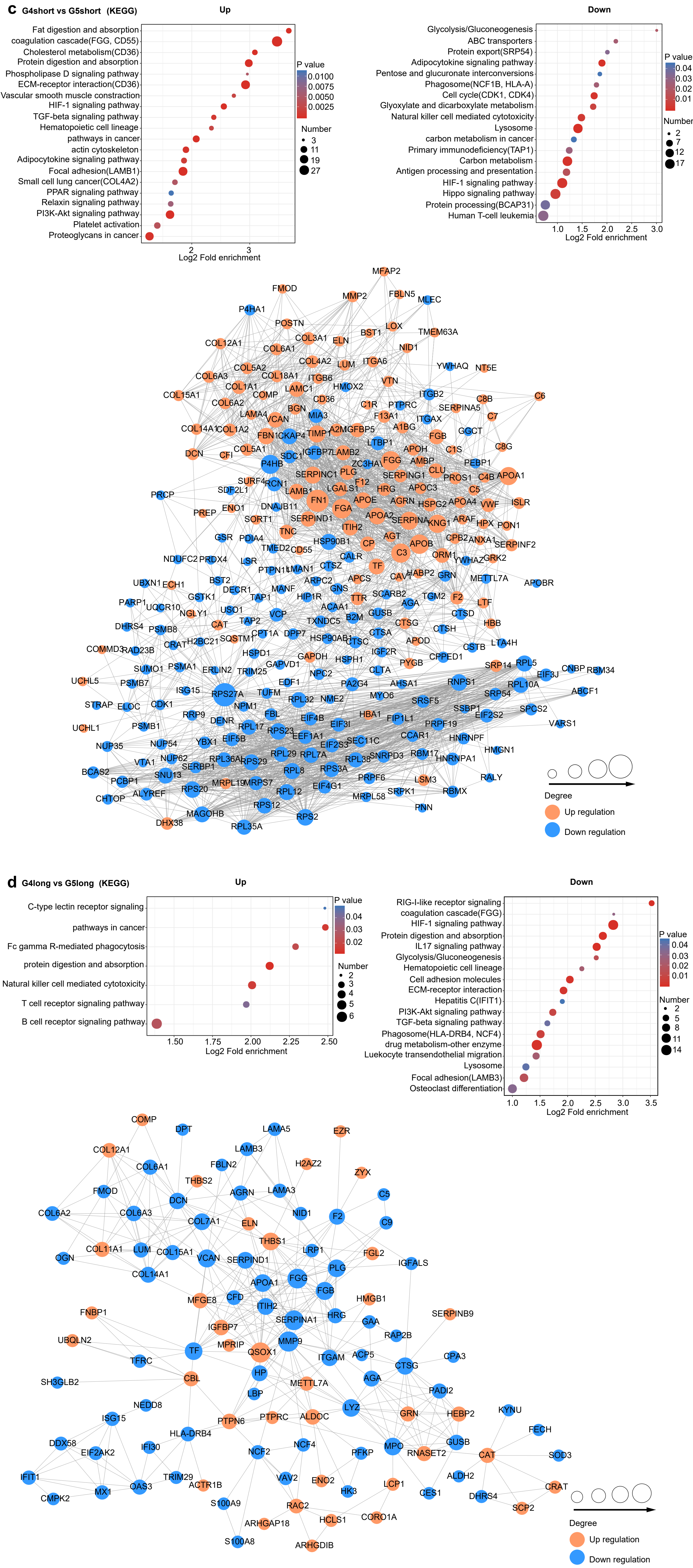

Supplementary Figure 7. Kyoto Encyclopedia of Genes and Genomes (KEGG) enrichment analysis of the differential pathways related to immune response. Different protein network based on global pattern analysis: Node size and color are based on betweenness centrality score of each node in the network. Top 10% of the most central nodes are indicated. (a) for G4short vs G4long, (b) for G5short vs G5long, (c) for G4short vs G5short and (d) for G4long vs G5long group. G4=Miller-Payne 4; G5=Miller-Payne 5.

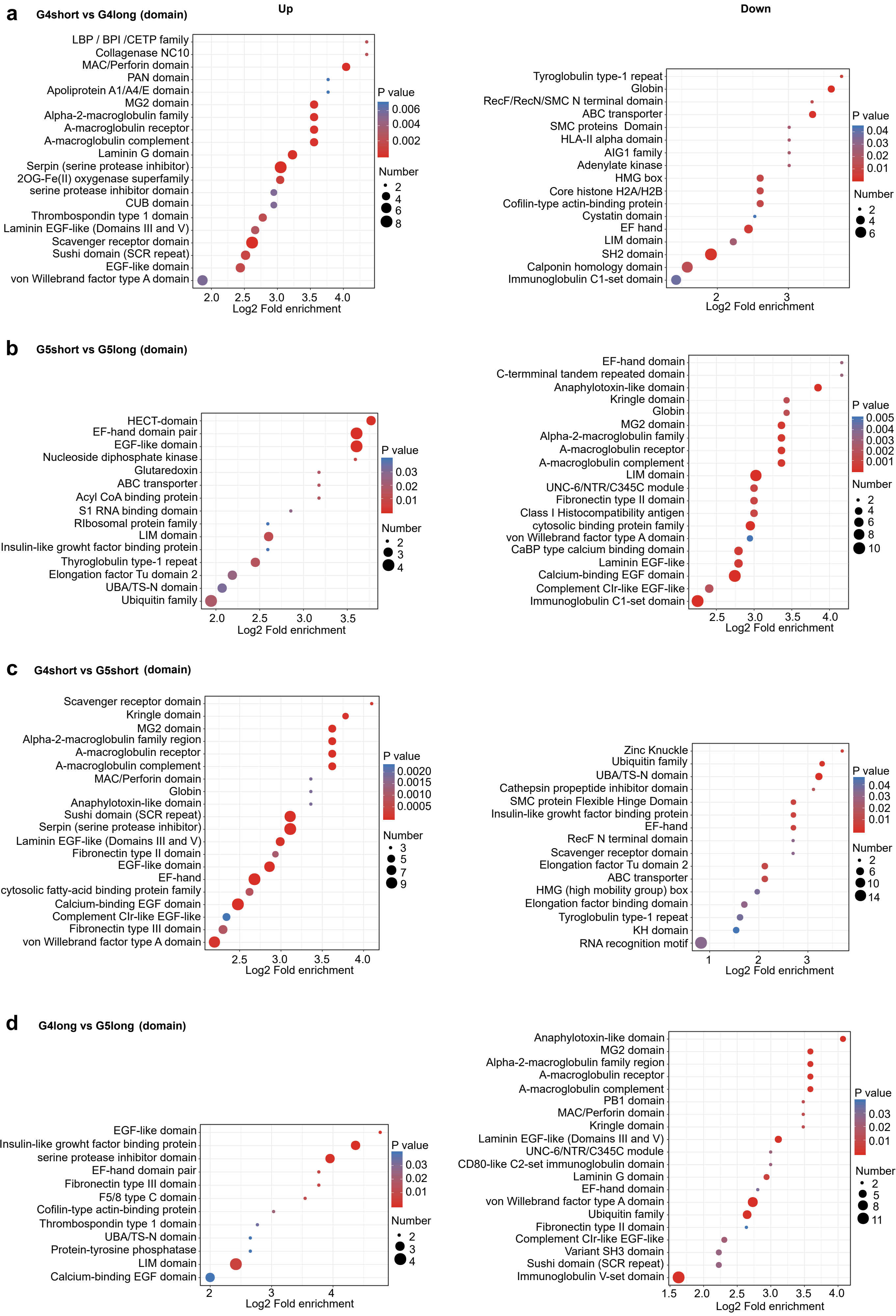

Supplementary Figure 8. Functional domain enrichment-based clustering analysis related to immune response in (a) for G4short vs G4long, (b) for G5short vs G5long, (c) for G4short vs G5short and (d) for G4long vs G5long group. Red represents a strong degree of enrichment, and blue represents a weak degree of enrichment. The size of point indicates the number of enriched proteins. G4=Miller-Payne 4; G5=Miller-Payne 5.

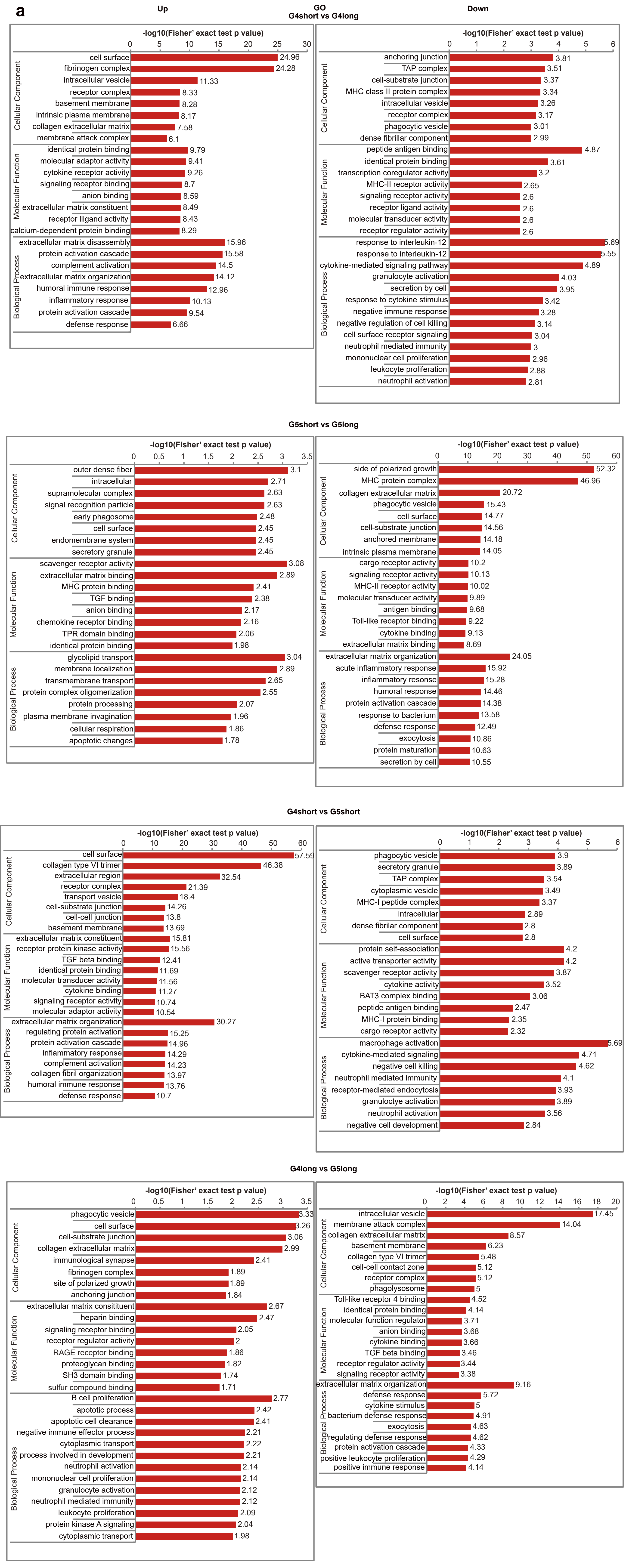

a
GO
Up
Down
G4short vs G4long
-log10(Fisher' exact test p value)
Cellular Component
cell surface 24.96
fibrinogen complex 24.28
intracellular vesicle 11.33
receptor complex 8.33
basement membrane 8.28
intrinsic plasma membrane 8.17
collagen extracellular matrix 7.58
membrane attack complex 6.1
Molecular Function
identical protein binding 9.79
molecular adaptor activity 9.41
cytokine receptor activity 9.26
signaling receptor binding 8.7
anion binding 8.59
extracellular matrix constituent 8.49
receptor ligand activity 8.43
calcium-dependent protein binding 8.29
Biological Process
extracellular matrix disassembly 15.96
protein activation cascade 15.58
complement activation 14.5
extracellular matrix organization 14.12
humoral immune response 12.96
inflammatory response 10.13
protein activation cascade 9.54
defense response 6.66
-log10(Fisher' exact test p value)
Cellular Component
anchoring junction 3.81
TAP complex 3.51
cell-substrate junction 3.37
MHC class II protein complex 3.34
intracellular vesicle 3.26
receptor complex 3.17
phagocytic vesicle 3.01
dense fibrillar component 2.99
Molecular Function
peptide antigen binding 4.87
identical protein binding 3.61
transcription coregulator activity 3.2
MHC-II receptor activity 2.65
signaling receptor activity 2.6
receptor ligand activity 2.6
molecular transducer activity 2.6
receptor regulator activity 2.6
Biological Process
response to interleukin-12 5.69
response to interleukin-12 5.55
cytokine-mediated signaling pathway 4.89
granulocyte activation 4.03
secretion by cell 3.95
response to cytokine stimulus 3.42
negative immune response 3.28
negative regulation of cell killing 3.14
cell surface receptor signaling 3.04
neutrophil mediated immunity 3
mononuclear cell proliferation 2.96
leukocyte proliferation 2.88
neutrophil activation 2.81
G5short vs G5long
-log10(Fisher' exact test p value)
Cellular Component
outer dense fiber 3.1
intracellular 2.71
supramolecular complex 2.63
signal recognition particle 2.63
early phagosome 2.48
cell surface 2.45
endomembrane system 2.45
secretory granule 2.45
Molecular Function
scavenger receptor activity 3.08
extracellular matrix binding 2.89
MHC protein binding 2.41
TGF binding 2.38
anion binding 2.17
chemokine receptor binding 2.16
TPR domain binding 2.06
identical protein binding 1.98
Biological Process
glycolipid transport 3.04
membrane localization 2.89
transmembrane transport 2.65
protein complex oligomerization 2.55
protein processing 2.07
plasma membrane invagination 1.96
cellular respiration 1.86
apoptotic changes 1.78
-log10(Fisher' exact test p value)
Cellular Component
side of polarized growth 52.32
MHC protein complex 46.96
collagen extracellular matrix 20.72
phagocytic vesicle 15.43
cell surface 14.77
cell-substrate junction 14.56
anchored membrane 14.18
intrinsic plasma membrane 14.05
Molecular Function
cargo receptor activity 10.2
signaling receptor activity 10.13
MHC-II receptor activity 10.02
molecular transducer activity 9.89
antigen binding 9.68
Toll-like receptor binding 9.22
cytokine binding 9.13
extracellular matrix binding 8.69
Biological Process
extracellular matrix organization 24.05
acute inflammatory response 15.92
inflammatory resonse 15.28
humoral response 14.46
protein activation cascade 14.38
response to bacterium 13.58
defense response 12.49
exocytosis 10.86
protein maturation 10.63
secretion by cell 10.55
G4short vs G5short
-log10(Fisher' exact test p value)
Cellular Component
cell surface 57.59
collagen type VI trimer 46.38
extracellular region 32.54
receptor complex 21.39
transport vesicle 18.4
cell-substrate junction 14.26
cell-cell junction 13.8
basement membrane 13.69
Molecular Function
extracellular matrix constituent 15.81
receptor protein kinase activity 15.56
TGF beta binding 12.41
identical protein binding 11.69
molecular transducer activity 11.56
cytokine binding 11.27
signaling receptor activity 10.74
molecular adaptor activity 10.54
Biological Process
extracellular matrix organization 30.27
regulating protein activation 15.25
protein activation cascade 14.96
inflammatory response 14.29
complement activation 14.23
collagen fibril organization 13.97
humoral immune response 13.76
defense response 10.7
-log10(Fisher' exact test p value)
Cellular Component
phagocytic vesicle 3.9
secretory granule 3.89
TAP complex 3.54
cytoplasmic vesicle 3.49
MHC-I peptide complex 3.37
intracellular 2.89
dense fibrilar component 2.8
cell surface 2.8
Molecular Function
protein self-association 4.2
active transporter activity 4.2
scavenger receptor activity 3.87
cytokine activity 3.52
BAT3 complex binding 3.06
peptide antigen binding 2.47
MHC-I protein binding 2.35
cargo receptor activity 2.32
Biological Process
macrophage activation 5.69
cytokine-mediated signaling 4.71
negative cell killing 4.62
neutrophil mediated immunity 4.1
receptor-mediated endocytosis 3.93
granulloctye activation 3.89
neutrophil activation 3.56
negative cell development 2.84
G4long vs G5long
-log10(Fisher' exact test p value)
Cellular Component
phagocytic vesicle 3.33
cell surface 3.26
cell-substrate junction 3.06
collagen extracellular matrix 2.99
immunological synapse 2.41
fibrinogen complex 1.89
site of polarized growth 1.89
anchoring junction 1.84
Molecular Function
extracellular matrix consitituent 2.67
heparin binding 2.47
signaling receptor binding 2.05
receptor regulator activity 2
RAGE receptor binding 1.86
proteoglycan binding 1.82
SH3 domain binding 1.74
sulfur compound binding 1.71
Biological Process
B cell proliferation 2.77
apoptotic process 2.42
apoptotic cell clearance 2.41
negative immune effector process 2.21
cytoplasmic transport 2.22
process involved in development 2.21
neutrophil activation 2.14
mononuclear cell proliferation 2.14
granulocyte activation 2.12
neutrophil mediated immunity 2.12
leukocyte proliferation 2.09
protein kinase A signaling 2.04
cytoplasmic transport 1.98
-log10(Fisher' exact test p value)
Cellular Component
intracellular vesicle 17.45
membrane attack complex 14.04
collagen extracellular matrix 8.57
basement membrane 6.23
collagen type VI trimer 5.48
cell-cell contact zone 5.12
receptor complex 5.12
phagolysosome 5
Molecular Function
Toll-like receptor 4 binding 4.52
identical protein binding 4.14
molecular function regulator 3.71
anion binding 3.68
cytokine binding 3.66
TGF beta binding 3.46
receptor regulator activity 3.44
signaling receptor activity 3.38
Biological Process
extracellular matrix organization 9.16
defense response 5.72
cytokine stimulus 5
bacterium defense response 4.91
exocytosis 4.63
regulating defense response 4.62
protein activation cascade 4.33
positive leukocyte proliferation 4.29
positive immune response 4.14

b

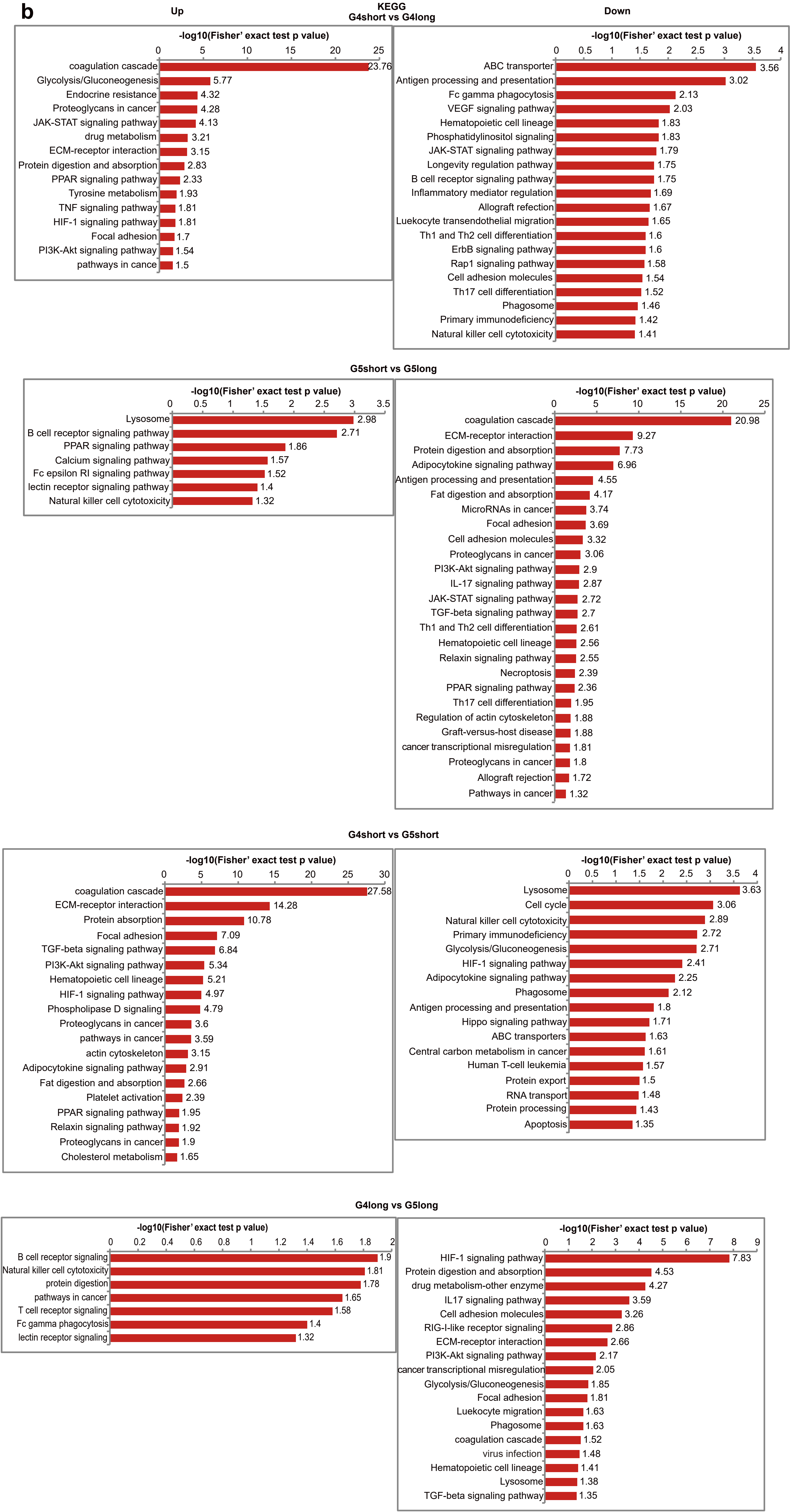
KEGG
G4short vs G4long
Up
Down
-log10(Fisher' exact test p value)
0 5 10 15 20 25
coagulation cascade 23.76
Glycolysis/Gluconeogenesis 5.77
Endocrine resistance 4.32
Proteoglycans in cancer 4.28
JAK-STAT signaling pathway 4.13
drug metabolism 3.21
ECM-receptor interaction 3.15
Protein digestion and absorption 2.83
PPAR signaling pathway 2.33
Tyrosine metabolism 1.93
TNF signaling pathway 1.81
HIF-1 signaling pathway 1.81
Focal adhesion 1.7
PI3K-Akt signaling pathway 1.54
pathways in cance 1.5
-log10(Fisher' exact test p value)
0 0.5 1 1.5 2 2.5 3 3.5 4
ABC transporter 3.56
Antigen processing and presentation 3.02
Fc gamma phagocytosis 2.13
VEGF signaling pathway 2.03
Hematopoietic cell lineage 1.83
Phosphatidylinositol signaling 1.83
JAK-STAT signaling pathway 1.79
Longevity regulation pathway 1.75
B cell receptor signaling pathway 1.75
Inflammatory mediator regulation 1.69
Allograft refection 1.67
Luekocyte transendothelial migration 1.65
Th1 and Th2 cell differentiation 1.6
ErbB signaling pathway 1.6
Rap1 signaling pathway 1.58
Cell adhesion molecules 1.54
Th17 cell differentiation 1.52
Phagosome 1.46
Primary immunodeficiency 1.42
Natural killer cell cytotoxicity 1.41
G5short vs G5long
-log10(Fisher' exact test p value)
0 0.5 1 1.5 2 2.5 3 3.5
Lysosome 2.98
B cell receptor signaling pathway 2.71
PPAR signaling pathway 1.86
Calcium signaling pathway 1.57
Fc epsilon RI signaling pathway 1.52
lectin receptor signaling pathway 1.4
Natural killer cell cytotoxicity 1.32
-log10(Fisher' exact test p value)
0 5 10 15 20 25
coagulation cascade 20.98
ECM-receptor interaction 9.27
Protein digestion and absorption 7.73
Adipocytokine signaling pathway 6.96
Antigen processing and presentation 4.55
Fat digestion and absorption 4.17
MicroRNAs in cancer 3.74
Focal adhesion 3.69
Cell adhesion molecules 3.32
Proteoglycans in cancer 3.06
PI3K-Akt signaling pathway 2.9
IL-17 signaling pathway 2.87
JAK-STAT signaling pathway 2.72
TGF-beta signaling pathway 2.7
Th1 and Th2 cell differentiation 2.61
Hematopoietic cell lineage 2.56
Relaxin signaling pathway 2.55
Necroptosis 2.39
PPAR signaling pathway 2.36
Th17 cell differentiation 1.95
Regulation of actin cytoskeleton 1.88
Graft-versus-host disease 1.88
cancer transcriptional misregulation 1.81
Proteoglycans in cancer 1.8
Allograft rejection 1.72
Pathways in cancer 1.32
G4short vs G5short
-log10(Fisher' exact test p value)
0 5 10 15 20 25 30
coagulation cascade 27.58
ECM-receptor interaction 14.28
Protein absorption 10.78
Focal adhesion 7.09
TGF-beta signaling pathway 6.84
PI3K-Akt signaling pathway 5.34
Hematopoietic cell lineage 5.21
HIF-1 signaling pathway 4.97
Phospholipase D signaling 4.79
Proteoglycans in cancer 3.6
pathways in cancer 3.59
actin cytoskeleton 3.15
Adipocytokine signaling pathway 2.91
Fat digestion and absorption 2.66
Platelet activation 2.39
PPAR signaling pathway 1.95
Relaxin signaling pathway 1.92
Proteoglycans in cancer 1.9
Cholesterol metabolism 1.65
-log10(Fisher' exact test p value)
0 0.5 1 1.5 2 2.5 3 3.5 4
Lysosome 3.63
Cell cycle 3.06
Natural killer cell cytotoxicity 2.89
Primary immunodeficiency 2.72
Glycolysis/Gluconeogenesis 2.71
HIF-1 signaling pathway 2.41
Adipocytokine signaling pathway 2.25
Phagosome 2.12
Antigen processing and presentation 1.8
Hippo signaling pathway 1.71
ABC transporters 1.63
Central carbon metabolism in cancer 1.61
Human T-cell leukemia 1.57
Protein export 1.5
RNA transport 1.48
Protein processing 1.43
Apoptosis 1.35
G4long vs G5long
-log10(Fisher' exact test p value)
0 0.2 0.4 0.6 0.8 1 1.2 1.4 1.6 1.8 2
B cell receptor signaling 1.9
Natural killer cell cytotoxicity 1.81
protein digestion 1.78
pathways in cancer 1.65
T cell receptor signaling 1.58
Fc gamma phagocytosis 1.4
lectin receptor signaling 1.32
-log10(Fisher' exact test p value)
0 1 2 3 4 5 6 7 8 9
HIF-1 signaling pathway 7.83
Protein digestion and absorption 4.53
drug metabolism-other enzyme 4.27
IL17 signaling pathway 3.59
Cell adhesion molecules 3.26
RIG-I-like receptor signaling 2.86
ECM-receptor interaction 2.66
PI3K-Akt signaling pathway 2.17
cancer transcriptional misregulation 2.05
Glycolysis/Gluconeogenesis 1.85
Focal adhesion 1.81
Luekocyte migration 1.63
Phagosome 1.63
coagulation cascade 1.52
virus infection 1.48
Hematopoietic cell lineage 1.41
Lysosome 1.38
TGF-beta signaling pathway 1.35

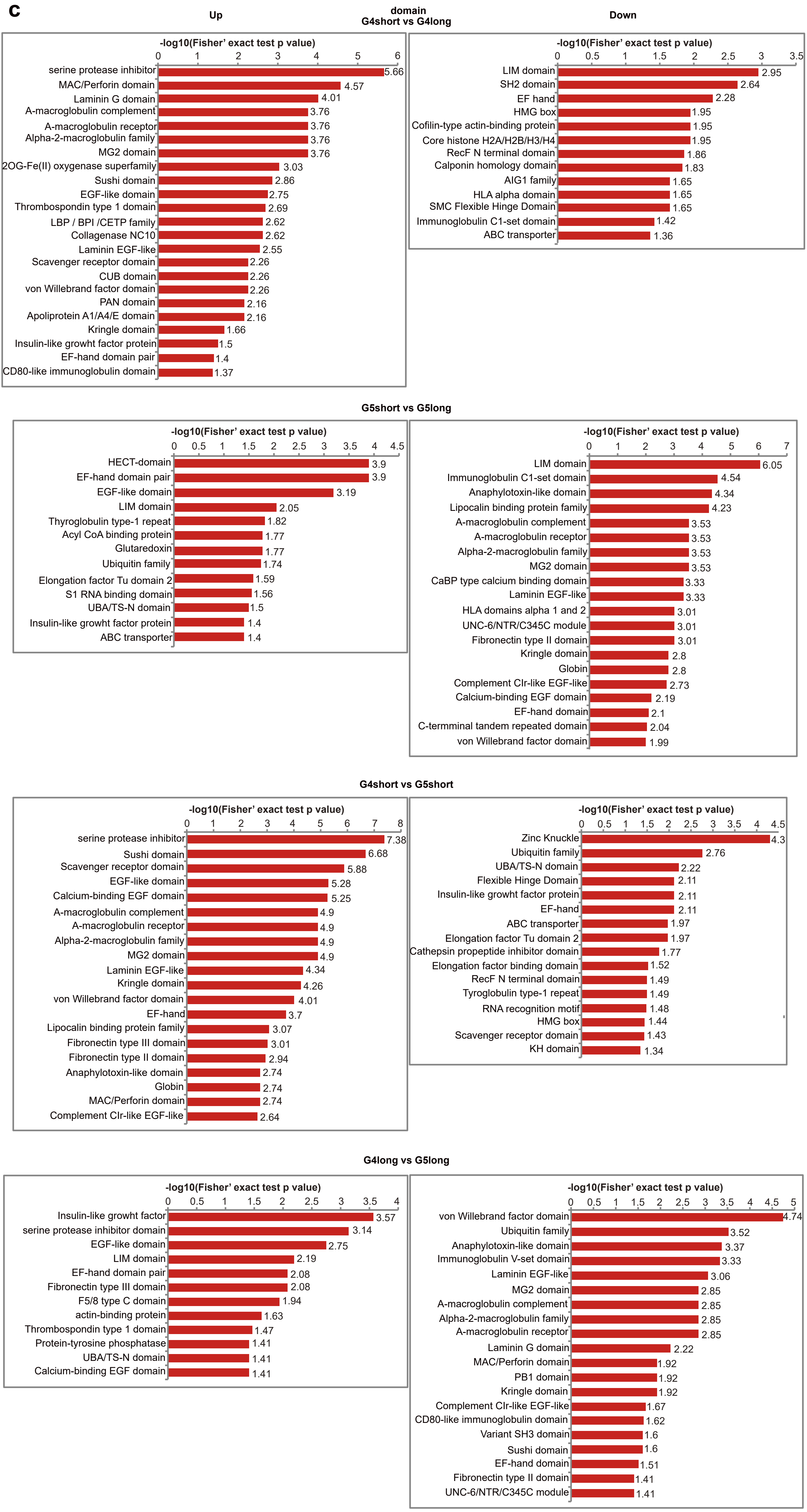

Supplementary Figure 9. Horizontal bar chart depicting significantly enriched canonical (a) GO classification, (b) KEGG and (c) functional domain of immune-related proteins by Fisher's exact test, in G4short vs G4long, G5short vs G5long, G4short vs G5short and G4long vs G5long group. G4=Miller-Payne 4; G5=Miller-Payne 5.

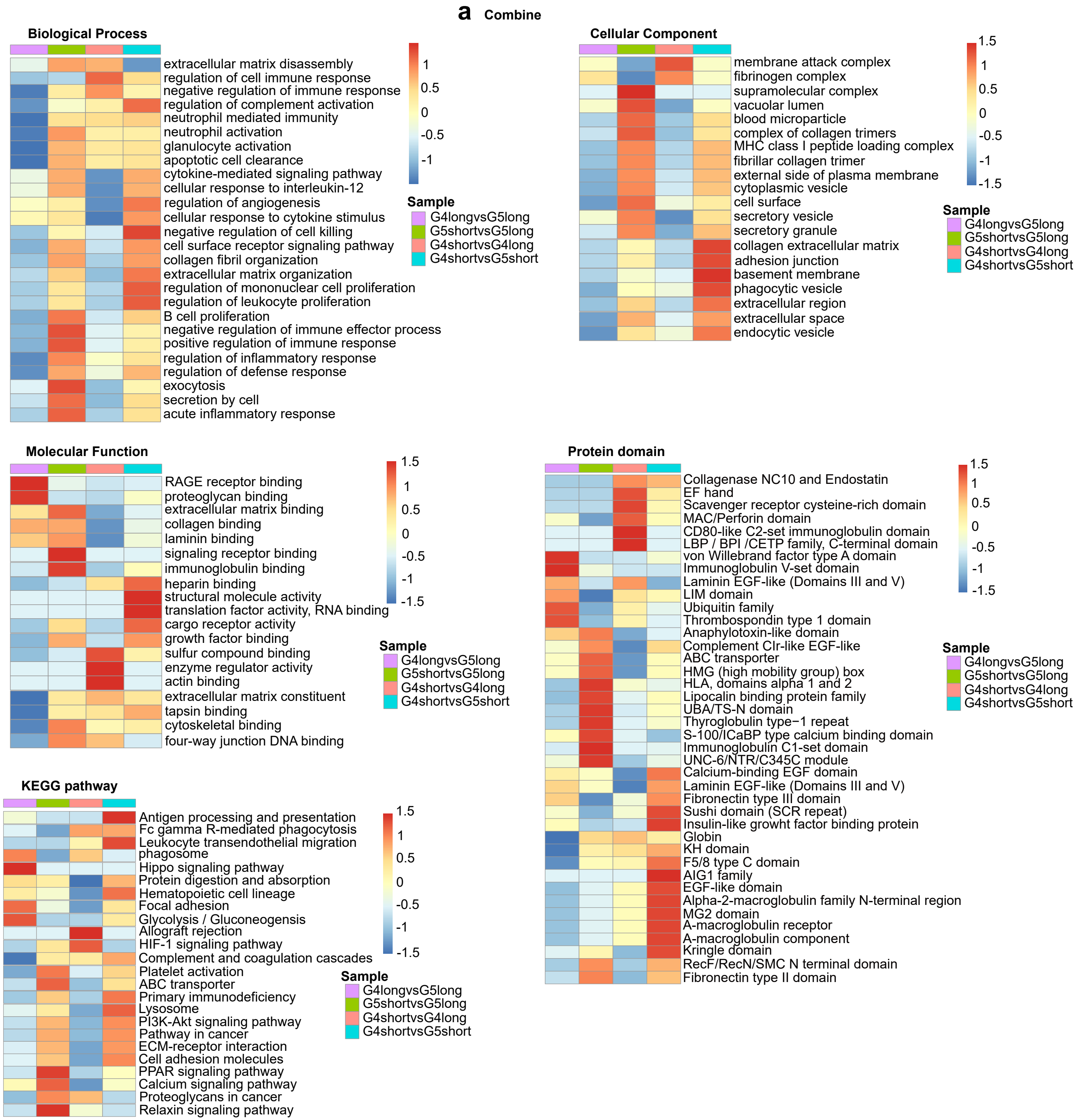
a Combine
Biological Process
extracellular matrix disassembly
regulation of cell immune response
negative regulation of immune response
regulation of complement activation
neutrophil mediated immunity
neutrophil activation
glanulocyte activation
apoptotic cell clearance
cytokine-mediated signaling pathway
cellular response to interleukin-12
regulation of angiogenesis
cellular response to cytokine stimulus
negative regulation of cell killing
cell surface receptor signaling pathway
collagen fibril organization
extracellular matrix organization
regulation of mononuclear cell proliferation
regulation of leukocyte proliferation
B cell proliferation
negative regulation of immune effector process
positive regulation of immune response
regulation of inflammatory response
regulation of defense response
exocytosis
secretion by cell
acute inflammatory response
1
0.5
0
-0.5
-1
Sample
G4longvsG5long
G5shortvsG5long
G4shortvsG4long
G4shortvsG5short
Cellular Component
membrane attack complex
fibrinogen complex
supramolecular complex
vacuolar lumen
blood microparticle
complex of collagen trimers
MHC class I peptide loading complex
fibrillar collagen trimer
external side of plasma membrane
cytoplasmic vesicle
cell surface
secretory vesicle
secretory granule
collagen extracellular matrix
adhesion junction
basement membrane
phagocytic vesicle
extracellular region
extracellular space
endocytic vesicle
1.5
1
0.5
0
-0.5
-1
-1.5
Sample
G4longvsG5long
G5shortvsG5long
G4shortvsG4long
G4shortvsG5short
Molecular Function
RAGE receptor binding
proteoglycan binding
extracellular matrix binding
collagen binding
laminin binding
signaling receptor binding
immunoglobulin binding
heparin binding
structural molecule activity
translation factor activity, RNA binding
cargo receptor activity
growth factor binding
sulfur compound binding
enzyme regulator activity
actin binding
extracellular matrix constituent
tapsin binding
cytoskeletal binding
four-way junction DNA binding
1.5
1
0.5
0
-0.5
-1
-1.5
Sample
G4longvsG5long
G5shortvsG5long
G4shortvsG4long
G4shortvsG5short
Protein domain
Collagenase NC10 and Endostatin
EF hand
Scavenger receptor cysteine-rich domain
MAC/Perforin domain
CD80-like C2-set immunoglobulin domain
LBP / BPI /CETP family, C-terminal domain
von Willebrand factor type A domain
Immunoglobulin V-set domain
Laminin EGF-like (Domains III and V)
LIM domain
Ubiquitin family
Thrombospondin type 1 domain
Anaphylotoxin-like domain
Complement Clr-like EGF-like
ABC transporter
HMG (high mobility group) box
HLA, domains alpha 1 and 2
Lipocalin binding protein family
UBA/TS-N domain
Thyroglobulin type−1 repeat
S-100/ICaBP type calcium binding domain
Immunoglobulin C1-set domain
UNC-6/NTR/C345C module
Calcium-binding EGF domain
Laminin EGF-like (Domains III and V)
Fibronectin type III domain
Sushi domain (SCR repeat)
Insulin-like growht factor binding protein
Globin
KH domain
F5/8 type C domain
AIG1 family
EGF-like domain
Alpha-2-macroglobulin family N-terminal region
MG2 domain
A-macroglobulin receptor
A-macroglobulin component
Kringle domain
RecF/RecN/SMC N terminal domain
Fibronectin type II domain
1.5
1
0.5
0
-0.5
-1
-1.5
Sample
G4longvsG5long
G5shortvsG5long
G4shortvsG4long
G4shortvsG5short
KEGG pathway
Antigen processing and presentation
Fc gamma R-mediated phagocytosis
Leukocyte transendothelial migration
phagosome
Hippo signaling pathway
Protein digestion and absorption
Hematopoietic cell lineage
Focal adhesion
Glycolysis / Gluconeogensis
Allograft rejection
HIF-1 signaling pathway
Complement and coagulation cascades
Platelet activation
ABC transporter
Primary immunodeficiency
Lysosome
PI3K-Akt signaling pathway
Pathway in cancer
ECM-receptor interaction
Cell adhesion molecules
PPAR signaling pathway
Calcium signaling pathway
Proteoglycans in cancer
Relaxin signaling pathway
1.5
1
0.5
0
-0.5
-1
-1.5
Sample
G4longvsG5long
G5shortvsG5long
G4shortvsG4long
G4shortvsG5short

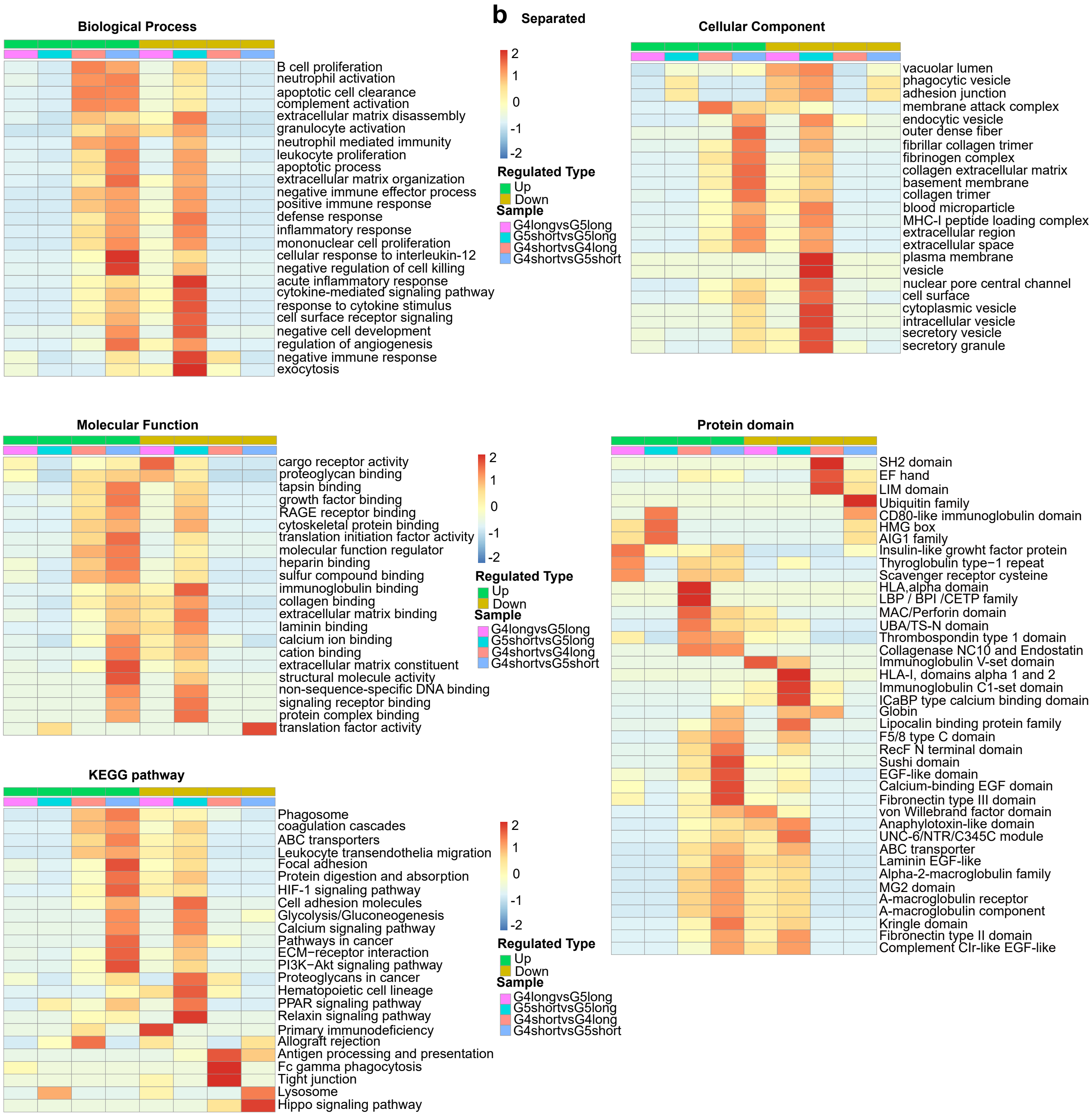

Supplementary Figure 10. Immune-related GO, KEGG pathways and functional domain are indicated (Fisher's exact test). (a) Comparison regarding the whole compared groups. (b) Comparison divided by up- and down-regulated degree within the compared groups. Red represents a strong degree of enrichment, and blue represents a weak degree of enrichment. G4=Miller-Payne 4; G5=Miller-Payne 5.

Supplementary dataset 1

1. Report document

Title	Number
Total spectrums	1298181
Matched spectrums	224302
Peptides	39700
Unique peptides	37061
Identified proteins	5161
Quantifiable proteins	3749

2. Basic analysis
3. Differentially expressed protein
4. Functional classification
5. Functional enrichment
6. PPI

All relative data were in EXCEL forms
