# Supplementary material for: Spatial immunophenotypes orchestrate prognosis in triple-negative breast cancer with Miller-Payne grade 4 following neoadjuvant chemotherapy
Source: NPJ Breast Cancer. 2023 Jul 12;9:57. doi: 10.1038/s41523-023-00565-8 (PMC10338497; doi:10.1038/s41523-023-00565-8)
Supplement: Supplementary file 3 — Reporting Summary [file 41523_2023_565_MOESM3_ESM.pdf]

nature portfolio

Corresponding author(s): Qingyuan Zhang

Last updated by author(s): Jun 9, 2023

Reporting Summary

Nature Portfolio wishes to improve the reproducibility of the work that we publish. This form provides structure for consistency and transparency in reporting. For further information on Nature Portfolio policies, see our Editorial Policies and the Editorial Policy Checklist.

Statistics

For all statistical analyses, confirm that the following items are present in the figure legend, table legend, main text, or Methods section.

n/a	Confirmed	
☐	☒	The exact sample size (*n*) for each experimental group/condition, given as a discrete number and unit of measurement
☐	☒	A statement on whether measurements were taken from distinct samples or whether the same sample was measured repeatedly
☐	☒	The statistical test(s) used AND whether they are one- or two-sided<br>*Only common tests should be described solely by name; describe more complex techniques in the Methods section.*
☐	☒	A description of all covariates tested
☐	☒	A description of any assumptions or corrections, such as tests of normality and adjustment for multiple comparisons
☐	☒	A full description of the statistical parameters including central tendency (e.g. means) or other basic estimates (e.g. regression coefficient) AND variation (e.g. standard deviation) or associated estimates of uncertainty (e.g. confidence intervals)
☐	☒	For null hypothesis testing, the test statistic (e.g. *F*, *t*, *r*) with confidence intervals, effect sizes, degrees of freedom and *P* value noted<br>*Give P values as exact values whenever suitable.*
☐	☒	For Bayesian analysis, information on the choice of priors and Markov chain Monte Carlo settings
☐	☒	For hierarchical and complex designs, identification of the appropriate level for tests and full reporting of outcomes
☐	☒	Estimates of effect sizes (e.g. Cohen's *d*, Pearson's *r*), indicating how they were calculated

Our web collection on statistics for biologists contains articles on many of the points above.

Software and code

Policy information about availability of computer code

Data collection	MIBI-TOF Imaging System (Fluidigm),timsTOF Pro (Bruker Daltonics) mass spectrometry instrument
Data analysis	MCDViewer, CellProfiler, and HistoCAT, PhenoGraph (v.2.0), The MaxQuant search engine (v.1.6.6.0),SPSS 25.0 version software (SPSS Inc. Chicago, USA) or GraphPad Prism 9

For manuscripts utilizing custom algorithms or software that are central to the research but not yet described in published literature, software must be made available to editors and reviewers. We strongly encourage code deposition in a community repository (e.g. GitHub). See the Nature Portfolio guidelines for submitting code & software for further information.

Data

Policy information about availability of data

All manuscripts must include a data availability statement. This statement should provide the following information, where applicable:

- Accession codes, unique identifiers, or web links for publicly available datasets
- A description of any restrictions on data availability
- For clinical datasets or third party data, please ensure that the statement adheres to our policy

We are willing to share all of the data and original code our report in our published paper with the research community. All relevant data are available from the authors. The mass spectrometry proteomics data have been deposited to the ProteomeXchange Consortium via the PRIDE partner repository with the dataset

identifier PXD029186. Username: reviewer_pxd029186@ebi.ac.uk
Password: 1KW52EcF

Research involving human participants, their data, or biological material

Policy information about studies with human participants or human data. See also policy information about sex, gender (identity/presentation), and sexual orientation and race, ethnicity and racism.

Reporting on sex and gender	For the breast, all the patients enrolled are female.
Reporting on race, ethnicity, or other socially relevant groupings	There are no race discrepancy. All the patients are Chinese from four clinical center of China.
Population characteristics	Patients characteristics have been summary in Table 1 and Table S2 as stated in manuscript in line 140. Please check.
Recruitment	Totally 272 patients were retrospectively reviewed and confirmed the diagnosis by experienced pathologists between January 2010 and January 2020. The prechemotherapy node negative status were firstly depended on clinical physical examination and ultrasound. For suspicious lesion/sample, hollow needle puncture or fine needle aspiration were conducted for pathological confirmation. Lacking more than 90% of tumor cells was defined as Miller-Payne 4 and no malignant cells identifiable in sections was Miller-Payne 5.
Ethics oversight	The study was performed in line with the Declaration of Helsinki and ethics approval was received from the institutional review board of these clinical centers. All participants provided written informed consent.

Note that full information on the approval of the study protocol must also be provided in the manuscript.

Field-specific reporting

Please select the one below that is the best fit for your research. If you are not sure, read the appropriate sections before making your selection.

☒ Life sciences ☐ Behavioural & social sciences ☐ Ecological, evolutionary & environmental sciences

For a reference copy of the document with all sections, see nature.com/documents/nr-reporting-summary-flat.pdf

Life sciences study design

All studies must disclose on these points even when the disclosure is negative.

Sample size	272 patients
Data exclusions	No data exclusions
Replication	No replications. Our study detect the clinical sample rather than the experiments in vitro or in vivo
Randomization	All the patients are randomly enrolled
Blinding	No blinding. This retrospective study should collect all the data from patients

Reporting for specific materials, systems and methods

We require information from authors about some types of materials, experimental systems and methods used in many studies. Here, indicate whether each material, system or method listed is relevant to your study. If you are not sure if a list item applies to your research, read the appropriate section before selecting a response.

Materials & experimental systems

n/a	Involved in the study	
☒	☐	Antibodies
☒	☐	Eukaryotic cell lines
☒	☐	Palaeontology and archaeology
☒	☐	Animals and other organisms
☒	☐	Clinical data
☒	☐	Dual use research of concern
☒	☐	Plants

Methods

n/a	Involved in the study	
☒	☐	ChIP-seq
☒	☐	Flow cytometry
☒	☐	MRI-based neuroimaging
